# Integrative approach for validation of six important fish species inhabiting River Poonch of north-west Himalayan region (India)

**DOI:** 10.3389/fgene.2022.1047436

**Published:** 2023-01-04

**Authors:** Mohd Awas, Imtiaz Ahmed, Syed Mudasir Ahmad, Khalid Mashay Al-Anazi, Mohammad Abul Farah, Basharat Bhat

**Affiliations:** ^1^ Fish Nutrition Laboratory, Department of Zoology, University of Kashmir, Srinagar, India; ^2^ Division of Animal Biotechnology, Faculty of Veterinary Sciences and Animal Husbandry, Sher-e-Kashmir University of Agricultural Sciences and Technology, Srinagar, India; ^3^ Department of Zoology, College of Science, King Saud University, Riyadh, Saudi Arabia

**Keywords:** fish diversity, Poonch River, *Cyt b*, 16S rRNA, validation, identification

## Abstract

Traditionally, species of fish are identified based on morphological characteristics. Although these taxonomic descriptions are essential, there are cases where the morphological characters distinguishing these species show marginal differences. For instance, in the Poonch River in the Himalayas, there are 21 species, out of which some are morphologically similar, and the taxonomic distinction between these species is unclear. Therefore, in this study, we used sequences from two mitochondrial genes, Cytochrome b (*Cyt b*) and a larger ribosomal subunit (16S rRNA), as well as the morphological analysis to address any taxonomic ambiguities among the six fish species. Maximum Likelihood results revealed that all the species were clustered according to their families and genera. The phenotypic analysis also supported this statement, as all the species of different genera like *Schizothorax*, *Tor*, *Garra*, *Traqilabeo*, and *Glyptothorax* are grouped in their particular cluster, it shows that species of a separate class share a mutual morphological characteristic. While genetic analyses of these species suggest nucleotide diversity (p) and haplotype diversity, with Hd values as 0.644 for *Cyt b* and 0.899 for 16S rRNA, confirming the rich genetic diversity in the river. Overall, we recommend that the integrative approach in delimiting the fish species is more effective than the individual one and can be used to rapidly diagnose a species and understand the evolutionary relationship between the species.

## Introduction

Over the last few decades, fish consumption has gradually become an important source of protein (FAO, 2000). The importance of fish as a source of human nutrition cannot be overstated because it contains high-quality protein, minerals and lipids high in omega-3 unsaturated fatty acids (Bavinck and Johnson, 2008), thus considered one of the most valuable components of the human diet. Besides, fisheries are important sources of revenue for many communities (Rafique and Khan, 2012; Ghouri et al., 2020). Over 33,000 species of fish are known, which inhabit nearly all major aquatic habitat types and contribute a wide range of environmental functions (Martinez et al., 2018). Around 2,500 fish species harbour Indian water and 930 are freshwater fish species (Jayaram, 2010). In India, 788 fish species come under freshwater fishes (Gopi, 2014). At the same time, the Himalayan region contributes about 17% of fish documented from the mountain ecosystem (Ghosh, 1997). In the present study, we have focused on the Poonch River of the north-west Himalayas, which originates from the foothill of Pir-panjal (Ahmed et al., 2019; Awas et al., 2020). This region is known for harboring diverse aquatic fauna, including 21 fish species, out of which six are considered economically important species (Hussain and Dutta, 2016).

Dutta (2003) reported 40 fish species from the Poonch River, including several species having sub-species under the same genus as *Schizothorax*, *Tor*, *Garra*, *Crossocheilus*, and *Glyptothorax*, etc. Such a considerable variation between these statements might be due to species ambiguity. Moreover, several findings suggest a vide ambiguity in *Schizothorax* and *Tor* genus (He and Chen, 2006; Yang et al., 2012; Ahmad et al., 2014; Dwivedi et al., 2020; Jaafar et al., 2021). However, in the region, these species under two genera are mainly considered as *Schizothorax richardsonii* (Luss) and *Tor putitora* (Chidak) due to their similarities in morphological traits. While Hussain and Dutta (2016) reported only *S. richardsonii* and *T. Putitora* belongs to the two genera from the Poonch River. Similarly, *Garra gotyla*, *Garra lamta*, *Tariqilabeo latius* and *Tariqilabeo diplocheius* have been reported by Dutta (2003) under four different species of two genera. But, due to the similarities in external morphological characteristics, there is a huge ambiguity among these species also, as Hussain and Dutta (2016) reported three species of two genera from this region.

To avoid mislabeling in fish markets, fish must be identified authentically and reliably. Morphological and morphometric characterization of fish species is one of the most often used strategies for fish authenticity (Bottero and Dalmasso, 2011). But limitations of classical taxonomy led to ambiguous categorization of fishes in the event of very close similarities between species and ultimately give an inaccurate picture of the ichthyofaunal diversity of the area (Gonzalez et al., 2013; Xu et al., 2018). Nowadays specific molecular techniques have been employed to overcome the constraints of morphological-based identification systems in fish (Hebert et al., 2003; Griffiths et al., 2013; Keskin and Atar, 2013; Zhuang et al., 2013; Knebelsberger et al., 2014; Diaz et al., 2016; Shen et al., 2016; Ghouri et al., 2020).

For fish identification, many DNA biomarkers have also been used. DNA barcoding has widely been used in different sectors, including fish authentication, labeling, biodiversity, conservation and biological studies (Pollack et al., 2018). The Cytochrome b (*Cyt b*) gene, which has ubiquitous uses in taxonomic and ecological domains, is one of the most well-known and targeted DNA markers. It is often used to study the phylogenetic relationships among organisms due to its small size and high nucleotide substitution rate (Xiao et al., 2001; Kumar et al., 2011; Ahmad et al., 2014; Cornetti et al., 2019). In addition, 16S ribosomal RNA has gained popularity due to its extremely high degree of conservation and relatively slow evolution rate (Hebert et al., 2003; Chen et al., 2018). Several past studies have shown that the 16S rRNA gene sequence is also helpful for species identification (Kochzius et al., 2010; Jaafar et al., 2021).

Most fish species inhabiting the Poonch River belong to the family Cyprinidae, represented by a group of species with highly similar external morphological characters. The survival of *Glyptothorax kashmirensis* (Kashmiri catfish) and *T. Putitora* (Mahaseer) exclusively depends on this water source. However, in 2010 Poonch River gained much more attention and was designated as a national park (Brown et al., 2019), but the lack of study based on the fish fauna is still marginal. Therefore, the fish species that have been recorded in the Poonch River; six have not been clearly classified by previous studies based on the analysis of morphological characters. To identify and classify these species unambiguously, we conducted this study using an integrative approach based on analyzing several morphological parameters and molecular research with *Cyt b* and 16S rRNA as genetic markers. The morphometric parameters used during the study were according to Froese and Pauly (2007). The different methods like PCA, CVA, RDA, etc. were used to get a more robust result; moreover, Jayaram (2010) standard keys were used for species determination. Besides, we also determine the efficacy of the selected genes in identifying the freshwater fish fauna. This study could be useful in examining DNA sequences, which might serve as a primary reference for future region studies.

## Materials and methods

### Fish sample collection

**FIGURE 1:**
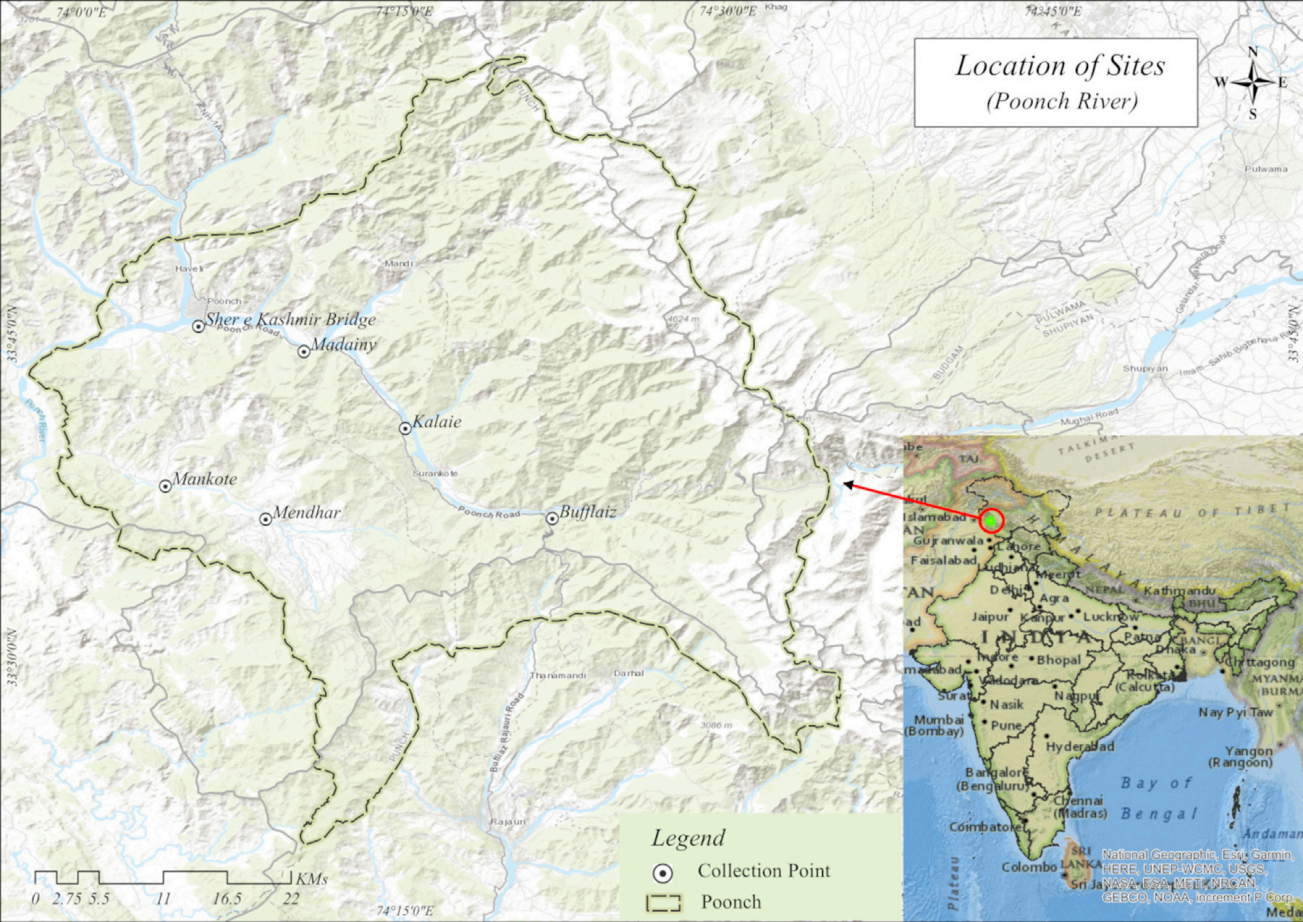
Map showing six sampling sites of River Pooch of north-west Himalaya India: Site A, Bufflaiz, B, Madainy, C, Kalai, D, Sher e Kashmir, E, Mendhar, F, Mankote.

In the present study, 478 specimens were sampled from 2018 to 2020 from six different sites in Poonch River with the help of native professional fishermen by using different fishing gears like cast net, gillnet and hooks, while the details about the number of samples collected, sampling sites and geo-graphical coordinates of locations for fish species investigated in the current study is provided in Table 1 and Figure 1, and the representative images of each species has shown in Supplementary Figure S1. All these specimens belonging to six species were carried out in an ice box at the Wet Laboratory, Department of Zoology, the University of Kashmir, for further study.

**TABLE 1:** Sampling sites, their locations with latitude and longitude, sample size and size range of six fish species used in the present study with GenBank accession number.

Species	Standard length (SL) range (cm)	Sample size		Sampling sites	Location	Cyt b	16S rRNA	IUCN status
		Traditional morphometrics	Molecular analysis					
*G. gotyla*	12.2–18.5	79	9	Madainy	33°40′42.57″N–74°15′7.35″E	MW191577, MW191578	MW148586, MW148585	LC
				Kalai	33°44′20.57″N–74°1′36.04″E			
				Bufflaiz	33°6′13.05″N–74°35′9.28″E			
				Mankote	33°37 ′ 59.14″ N–74°3′54.02″ E			
*T. putitora*	17.1–28.8	77	8	Mendhar	33°36′24.07″N–74°8′36.74″E	MW191579, MW191580, MW191581	MW148589	EN
				Sher-e-Kashmir bridge	33°7′58.33″N–74°9′19.44″E			
				Mankote	33°37 ′ 59.14″N–74°3′54.02″E			
				Mendhar	33°36′24.07″N–74°8′36.74″E			
				Sher-e-Kashmir bridge	33°7′58.33″N–74°9′19.44″E			
				Mankote	33°37 ′ 59.14″N–74°3′54.02″E			
				Kalai	33°44′20.57″N–74°1′36.04″E			
*S. richardsonii*	10.32–31.76	79	8	Bufflaiz	33°6′13.05″N–74°35′9.28″E	MW191586	MW148587	VU
				Mendhar	33°36′24.07″N–74°8′36.74″E			
				Kalai	33°44′20.57″N–74°1′36.04″E			
				Sher-e-Kashmir bridge	33°7′58.33″N–74°9′19.44″E			
				Madainy	33°40′42.57″N–74°15′7.35″E			
*S. plagiostomus*	14.2–28.9	78	7	Madainy	33°40′42.57″N–74°15′7.35″E		MW148588	NA
				Sher-e-Kashmir bridge	33°7′58.33″N–74°9′19.44″E			
				Kalai	33°44′20.57″N–74°1′36.04″E			
				Bufflaiz	33°6′13.05″N–74°35′9.28″E			
*T. latius*	11.1–16.2	79	8	Madainy	33°40′42.57″N–74°15′7.35″E	MW191585	MW148582, MW148583, MW148584	LC
				Mankote	33°37 ′ 59.14″N–74°3′54.02″E			
				Kalai	33°44′20.57″N–74°1′36.04″E			
				Bufflaiz	33°6′13.05″N–74°35′9.28″E			
*G. kashmirinsis*	6.9–11.2	37	2	Bufflaiz	33°6′13.05″N–74°35′9.28″E		MW148582	CR
				Madainy	33°40′42.57″N–74°15′7.35″E			
				Sher-e-Kashmir bridge	33°7′58.33″N–74°9′19.44″E			

### Identification

The collected fish species were first identified using standard taxonomic keys and related books (Talwar and Jhingran, 1991; Vishwanath et al., 2007; Jayaram, 2010). After proper identification, representative specimens were preserved in 10% formalin in the Fish Nutrition Research Laboratory, Department of Zoology, University of Kashmir Srinagar, India, for museum records.

### Morphometric and meristic controlling elements

Different morphometric and meristic controlling elements were also taken across the fish body by following different methods reported in the past (Menon, 1964; Gonzales and Sartori, 2002; Mina et al., 2005; Froese and Pauly, 2007). The measurements were recorded using a 0.01 cm digital vernier caliper from left to right. Overall, 25 phenotypic characters were analyzed. Counts and measurements were taken as far as possible on the left side of fish specimens following standard methods for cyprinid taxonomy (Pethiyagoda, and Kottelat, 1995) with some additional modifications. The morphometric characters measured during the present study (Supplementary Figure S2) included total length (TL), standard length (SL), forked length (FL), pre-pectoral length (PpL), pre-pelvic length (ppL), pre-dorsal length (PDL), pre-anal length (PAL), pectoral fin length (PFL), pectoral fin height (PFH), pelvic fin length (pFL), pelvic fin height (pFH), dorsal fin length (DFL), dorsal fin height (DFH), anal fin length (AFL), anal fin height (AFH), caudal fin length (CFL), caudal fin height (CFH), head length (HL), snout length (SL), eye diameter (E-dia), pre-orbital length (PorbL), minimum body depth (MinBD), maximum body depth (MaxBD). While meristic characters were studied, including dorsal fin rays (DFR), pectoral fin rays (PFR), pelvic fin rays (pFR), anal fin rays (AFR), and caudal fin rays (CFR). Meristic characters were counted twice by the same observer. In the current study, it was hypothesized that a working relationship exists between various growth-related morphometric parameters. The correlation coefficient “r” was determined among all the characteristics.

### Traditional morphometric analysis

Each of the fourteen morphometric characters was divided by standard length and the remaining six characters were divided by head length in order to eliminate the size effect (correlation < .5 for all variables). All the morphometric values were log-transformed before analysis in R software (R Development Core Team, 2020). The regression analysis and the resulting figures were plotted by using “psych” (Wickham, 2016; Revelle, 2020).

### Statistical analysis

All statistical analyses were carried out using R Statistical Software (R Development Core Team, 2020) to perform all morphometric variation analyses among different fish species. We calculated the descriptive statistics for each morphometric parameter, particularly mean standard deviation, using the “dplyr” package (Wickham, et al., 2022). Prior to conducting statistical analysis, we used the Sharpio–Wilk normality test to investigate the normality assumption of the dataset. In order to determine if there were significant differences in morphometric characters, a univariate analysis of variance (ANOVA) was performed. In order to decrease the amount of sample redundancy, a principal component analysis (PCA) was conducted (Johnson and Wichern 1998). Before conducting PCA, the morphometric measurements were subjected to scaling (i.e., standardization) to make the analysis comparable. Moreover, we also evaluated the correlation of studied phenotypic characters with principal components analysis. In general, the variables which are correlated with PC1 or dimension (Dim, 1) and PC2 or Dim, 2 are considered as crucial in order to explain the existing variability among the data set (Kassambara, 2017). The PCA analysis and the resulting figures were obtained using “FactoMinerR” (Le et al., 2008) and “factoextra” (Kassambra and Mundt, 2020) packages. In addition to PCA, multiple variables were constructed and further analytical procedures, such as canonical variate analysis (CVA) and redundancy analysis (RDA), were conducted (Mardia et al., 1979; Ter Braak and Verdonschot, 1995). Moreover, the sample size was taken based on previous work on PCA and CVA (Kocovsky et al., 2009). The contributions of the variable to PCA were investigated to decide which character contributes more to differentiate the species. For this purpose, the Kaiser–Meyer–Olkin value (0–1) was used to check the suitability for PCA (Kaiser, 1974). Various multivariate analyses, such as PCA, CVA, RDA, and correlations, were performed in R. 4.0.2. However, the dendrogram was constructed to address the dissimilarity between species and the number of clusters in the data set (Charrad et al., 2014).

In order to avoid the effect of sample size, the data were M-transformed by employing the formula given below (Poulet et al., 2005).

$$M\;trans=\log\;M-b\;\left(\log\;SL-\log\;SL\;mean\right)$$

where M−trans is the transformed measurement, M is the original measurement, b is the within-group slope regression of the log standard length, SL is the standard length of the fish species. To assess the effectiveness of size adjustment transformations, the relevance of the relationship between transformed variables and standard length was taken into consideration. The correlation analysis between the modified variables and the fish’s standard length was determined to evaluate whether the transformed data successfully removed the size effect or not (Khan et al., 2012).

### Molecular analysis

For molecular analysis, all six species having morphological ambiguities were used to study the diversity and phylogenetic relationship. For barcoding purposes, 2–3 specimens of representative species were preserved in 95% alcohol (Roul et al., 2021; Tsoupas et al., 2022), as the *G. kashmirensis* is one of the protected species; therefore, a small part of the fin was preserved for DNA analysis before releasing the surviving fish back into the water. For further analysis, the samples were transferred to the Genomics Laboratory at Sher-e-Kashmir University of Agricultural Science and Technology, Jammu and Kashmir, India.

### DNA extraction and PCR amplification

Using a sterile razor blade, we extracted DNA from a portion of muscle tissue from the caudal peduncle. The muscle sample was placed in Eppendorf tubes, rinsed with 1 ml phosphate-buffered saline (PBS) in order to remove all contamination, and then the sample was frozen. After discarding (PBS), total genomic DNA was extracted using DNeasy Blood and Tissue Kit (QIAGEN 69504) by following the instruction given by the company. After that, the product was quantified with the help of NanoDrop (Thermo Scientific, Wilmington, NC, United States). Extracted genomic DNA was amplified for the above two genes using universal primers. The primers against 16S rRNA forward (5′ GCC TGT TTA ACA AAA ACA T 3′) and reverse (5′ CCG GTC TGA ACT CAG ATC ATG T3′) (Simon et al., 1991) and Cyt b forward (5′ GTT TGA TCC CGT TTC CTG TA 3′) and reverse (5′ AAT GAC TTG AAG AAC CACCGT 3′) were used for amplification (Briolay et al., 1998). Afterwards, a PCR reaction mixture was performed as per the protocol mentioned in the kit. The following steps were taken for PCR reaction. Initial denaturation for 3 min at 95°C, followed by 34 cycles of 95°C for 30 s (16S) after annealing at 48°C and 50°C for (*Cyt b*), while extension temperature of 72°C for 1 min and final extension of 72°C for 3 min. PCR products were visualized on 1% agarose gel. Finally, the product was purified using a PCR purification kit (Invitrogen, United States) using the company^’^s protocol. Biospectrometer measured the purified PCR products (Thermo Scientific, United States).

### DNA sequencing and data analysis

PCR amplified products were sequenced at genomic Xcelris Labs Limited Ist Floor, Sydney House, Old Premchand Nagar Road, Opp. Satyagrah Chhavani, Bodakdev, Ahmedabad—380015 Gujarat, India. The specific primers successfully amplified the *Cyt b* and 16S rRNA genes of the approximate size of 543 and 519 bp, respectively. The data was aligned with the help of MUSCLE v3.5 (Edgar, 2004) and the ClustalW algorithm (50), whereas the bio-editing tool was done using MacClade v4.06 (Maddison and Maddison, 2003). All the ambivalent bases were removed with the help of ABI chromatograms and the rest of the sequences were submitted to GenBank to get accession numbers. The phylogenetic trees were constructed with the help of PhyML algorithm software using the Maximum likelihood method (Felsenstein, 1981), with jModelTest (Posada, 2008; Darriba et al., 2012) and applied the bootstrap technique set to 100 replicates for testing the trees (Guindon et al., 2009). *Barbus barbus* was used as the out-group. Automatic Barcode Gap Discovery (ABGD) clustering was carried out using Kimura evolutionary model available at (https://bioinfo.mnhn.fr/abi/public/abgd/abgdweb.html) by applying the following parameter: Pmin = .001, Pmax = .1; steps 20; Nb bins = 20; X (gap width) = .75 and Nb bins (for distance distribution) = 20 (Puillandre et al., 2012). By using pairwise distances from the sequence and the prior intra-specific divergence, ABGD analysis detects the gap between intra and interspecific divergence.

The DNA Sequence Polymorphism 6.12.03 software was used to measure nucleotide diversity (p) and haplotype diversity (Hd) in order to analyze genetic diversity in the population of six different locations from Poonch River in a single data set that may help to predict genetic diversity within the population of six endemic fish species found in the region. The first dataset included four species belonging to the family Cyprinidae for the analysis of *Cyt b*, and five species belonging to two families were examined through 16S rRNA in the second subset. While in the third subset, combined studies were performed for both the genes for all the individuals.

## Results

### Phenotypic analysis/identification

**FIGURE 2:**
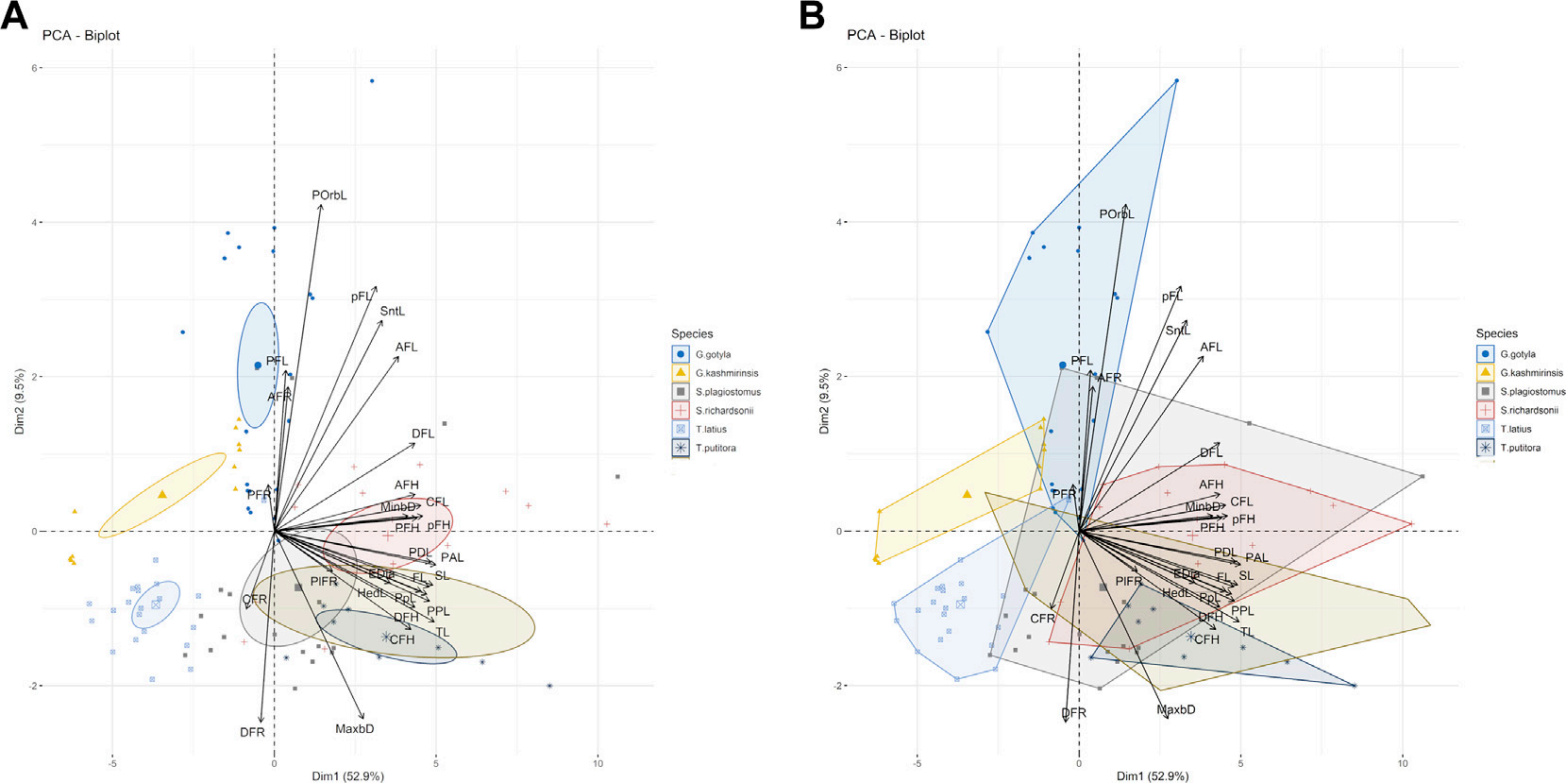
**(A,B)** Bi-Plot the variables and species oriented along the first two principal components with species. The Dim 1 and Dim 2 are the first and second principal extracted in PCA and notation in graph refers to the morphometric variables.

**FIGURE 3:**
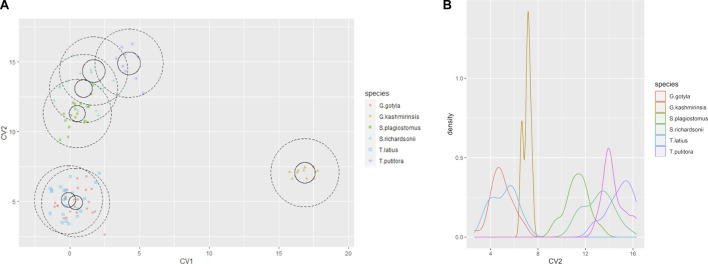
**(A)** The Canonical Variate (CVA) showing the morpho-genetic variation of six fish species collected from River Poonch **(B)** biplot of same centroids of the canonical variate score are shown on the 25 morphometric distances.

**FIGURE 4:**
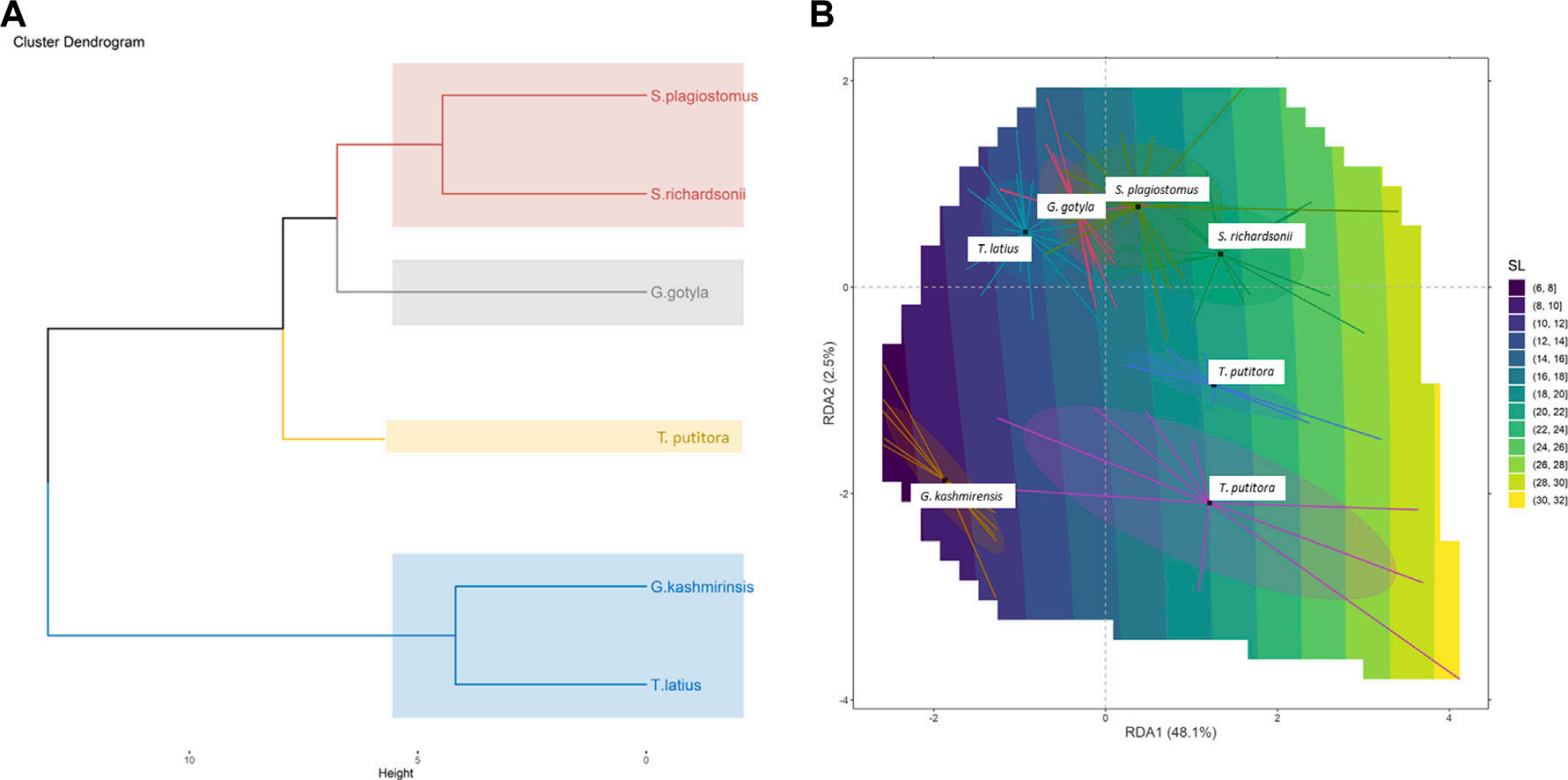
**(A)** Redundancy analysis (RDA) Biplot showing taxonomic division among six fish species collected from River Poonch. **(B)** Cluster dendrogram derived from morphometric analysis.

The current study successfully identified six endemic freshwater fish species from the Poonch River of the Northwest Himalayan range of J&K (India), i.e., *S. richardsonii*, *Schizothorax plagiostomus*, *T. putitora*, *G. gotyla*, *T. latius* and *G. kashmirensis*. The species belong to two families and two orders. As per the International Union for Conservation of Nature (IUCN), 2018 status, out of six identified species, *G. kashmirensis* is critically endangered (CR), *T. putitora* endangered (EN), *S. richardsonii* enlisted as vulnerable (VU), *S. plagiostomus* not evaluated (NA), while *G. gotyla* and *T. latius* are listed as least concerned (LC), the same is presented in Tables 1, 2. Multivariate and cluster analyses like ANOVA, PCA, CVA, RDA, dendrogram, and molecular approaches like ABGD, BI, and Phylogenetic Trees were employed to get a robust result. ANOVA demonstrates a significant (F = 20.90, *p* < .05) difference in phenotypic measurements and relations in the present study. It is found that by performing the principal component analysis (PCA), 28 principal components were extracted, of which the first two (PC1 and PC2) differentiated 62.4% of the entire variation in terms of variance (52.9 for PC1, and 9.5% for PC2). In terms of influence variables on PC1 the PCA components with the highest loadings are (Total length (TL), Standard length (SL), Forked length (FL), Pre-pectoral length (PPL), Pre-pelvic length (PpL), Pre-dorsal length (PDL), Pre-Anal length (PAL), Pectoral fin height (PFH), pelvic fin height (pFH), Dorsal fin length (DFL), Dorsal fin height (DFH), Anal fin length (AFL), Anal fin height (AFH), Caudal fin length (CFL), Caudal fin height (CFH), Eye diameter (EDia), Head length (HdL), Maximum body depth (MaxbD), Minimum body depth (MinbD). Correspondingly, in PC2, the most crucial loading was Pectoral Fin length (PFL), Pelvic Fin length (pFL), Snout Length (SntL), Pre-Orbital length (PorbL), Dorsal Fin rays (DFR), Anal Fin rays (AFR) and Caudal Fin rays (CFR). The Biplot revealed that all stocks were mixed and overlapped, as shown in Figures 2A, B. According to the PCA plot of PC1 and PC2 (Figure 2B), there is a high overlap between two species of Schizothorax genera. However, morphological variation occurred even in *T. putitora* collected during different season. Species such as *G. kashmirensis* maintained clear differentiation from others. On the whole, PCA separated fish species based on the variation in their outline, regardless of their size, with high overlaps among strictly associated species. As a result of high levels of overlap between groups, further verification was essential through CVA to obtain shape variations. There were 28 canonical variates that accounted for all variances. In the analysis of variance, the first two canonical variables (CV1 and CV2) differentiated 73.68% of the total difference. The importance of CV1 was 45.09% and the importance of CV2 was 28.59%. CVs from two closely related species with overlapping body shapes were observed in the first two CVs (Figures 3A, B). In CVA plots, *T. putitora* collected from different season and *Schizothorax* genera overlapped highly, indicating a high degree of similarity in their shapes and phenotypic characteristics. However, the *G*. *kashmirinsis* species was placed distinctly from other species groups as this fish belongs to different families. Similar results were obtained through RDA, in which five species were separated at RDA1 and one species (*G. kashmirinsis*) were differentiated at RDA2, as shown in Figure 4A. Moreover, the phenotypic results were conformed through a dendrogram in which all six species were separated successfully shown in Figure 4B.

**TABLE 2:** Comparative analysis of some important phenotypic characters of six fish species habiting River Poonch with already published data.

Morphometric/meristic character		*S. richardsonii*	*S. plagiostomus*	*T. putitora*	*G. gotyla*	*T. latius*	*G. kashmiriensis*
Standard length	Present study	10.32–31.76 ± 3.2	14.2–28.9 ± 3.47	17.1–28.8 ± 3.7	12.2–18.5 ± 1.50	11.1–16.2 ± 1.67	6.9–11.2 ± 1.67
	Earlier reported	19.85 ± 3.25, 15.55–19.06	12.4–32.9 ± 3.1	16.4–24.2 ± 5.5, 14–32	12.1 ± 1.6, 6.6–11.9, 12.1 ± 1.6	7.55–13.50 ± 1.93, 7.55–13.50	9.2
	References	Khan et al. (2020). Mehmood et al. (2021), Regmi et al. (2021); Lohani et al. (2020)	Mir et al. (2013a); Bhat et al. (2013)	Kamboj & Kamboj, (2019); Mehmood et al. (2021)	Brraich & Akhter, (2015); Attaullah et al. (2021)	Sharma et al. (2014); Mir et al. (2013b)	Wahab and Yousafzai (2017)
Maximum body depth	Present study	3.8–7.5 ± 1.6	2.9–6.9 ± 1.8	2.9–6.9 ± 1.7	1.5–4.9 ± 1.34	3.1–4.7 ± 0.43	2.6–3.7 ± 0.43
	Earlier reported	4.95 ± 1.08, 2.69–4.21	2.7–5.87 ± 1.18	4.1–7.5 ± 1.4, 3.8–8.2	1.7–3.6, 2.2 ± 0.1	1.91–2.92 ± 0.38	2.1
Minimum body depth	Present study	1.9–3.5 ± 0.7	1.6–3.9 ± 1.4	2.1–3.8 ± 0.6	1.7–2.5 ± 1.04	1.3–2.9 ± 0.76	1.3–2.3 ± 0.76
	Earlier reported		1.45–3.23	1.3–4	1–1.9	0.90–1.70 ± 0.22	
Head length	Present study	3.3–5.9 ± 0.6	2.9–5.7 ± 1.06	4.2–7.1 ± 0.9	2.9–3.7 ± 0.81	2.5–3.2 ± 0.19	2.5–3.2 ± 0.19
	Earlier reported	4.75 ± 0.45	2.87–4.98	3.5–6.9	2–3.8 ± 1.8	1.83–2.50 ± 0.22, 1.83–2.50	2.5
Dorsal fin rays	Present study	6–9	6–9	8–12	6–9	8–10	8–10
	Earlier reported	8.25 ± 0.48	9.38 ± 0.21	12 (3/9), 9–13	6–10	III-8	
Pectoral fin rays	Present study	12–16	12–16	14–19	12–14	12–14	12–14
	Earlier reported	18.00 ± 0.91	15.31 ± 0.61	19, 10–15	12–14 (1–6/8–12)	I-13	
Pelvic fin rays	Present study	9–11	9–11	8–9	8–9	8–9	8–9
	Earlier reported	11.50 ± 0.65	11.23 ± 0.39	9	8–9	I-8	
Anal fin rays	Present study	5–6	5–7	5–6	6–7	5–6	5–6
	Earlier reported	7.75 ± 0.85	7.00 ± 0.23	2–6, 5–8	6 (1–2/4–5)	I-5	
Caudal fin rays	Present study	16–18	16–18	16–19	16–19	16–20	16–20
	Earlier reported	17.9 ± 2.87	18.00 ± 0.23	19, 15–22	17–19 (2–5/13–17)	II-17–18	

**FIGURE 5:**
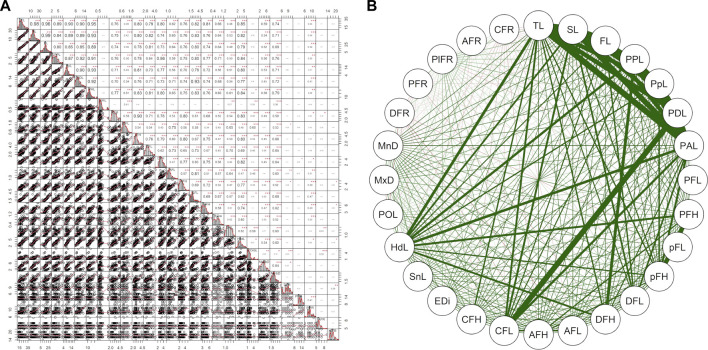
**(A)** The correlation plot among the morphometric parameter among six fish species collected from River Poonch. The value given around all the axes are the range of each species parameter s measured unit values (cm). Correlation coefficients (r) are indicated with numeric values, with significant levels (p) are denoted by asterisks (* < .05, ** < .01, *** < .001) and **(B)** presenting same in diagrammatic value.

The correlation plot in Figures 5A, B and Supplementary Tables S2, S4–S6 depicted that all phenotypic characters varied uniformly in total length and provided significant and strong positive correlations. The result revealed Head length (HdL), Anal fin length (AFL), Total length (TL), Standard Length (SL), Forked length (FL), Pre-pectoral length (PPL), Pre-pelvic length (PpL), Pre-Dorsal length (PDL), Pre-Anal length (PAL) and Caudal Fin length (CFL) are the most important character that plays a key role in the identification of these fish species in which the value if *p* is < .001. The highest correlation for *T. putitora* with reverence to total length (Supplementary Tables S1, S3) was observed for forked length (*r* = .99), pre-anal length (*r* = .99), standard length (*r* = .98), pre-pelvic length (*r* = .98), whereas lowermost was recorded for pelvic fin length (*r* = 52) and caudal fin height (*r* = .55). In the case of head length for *T. putitora*, pre-orbital length (*r* = .77) showed the lowest correlation for head length and the eye diameter (*r* = .52).

Moreover, the present study depicts the pelvic fin length as a diagnostic feature to differentiate *S. richardsonii* from *S. plagiostomus*. However, the highest correlation in overall size was observed for standard length (*r* = .98), forked length (*r* = .98), pre-pelvic fin length (*r* = .96), while the lowest correlation with maximum body depth (*r* = .58), pectoral fin length (*r* = 59). While about head length, the maximum was eye diameter (*r* = .67) and minimum pre-orbital length (*r* = .49) for *S. richardsonii*. The result obtained for *S. plagiostomus* as maximum correlation concerning total length with standard length (*r* = .99), forked size (*r* = .99) pre-pectoral size (*r* = .94), while the least was seen in the case of caudal fin length (*r* = .57), and maximum body depth (*r* = .66). The highest correlation was observed in head length for pre-orbital length (*r* = .52), and the lowest was eye diameter (*r* = .44), as shown in Supplementary Tables S2, S3, S9.

### Molecular analysis

Integrative methods successfully identified all the unpublished *Cyt b* and 16S rRNA sequences from six fish species as it was determined that there was no mismatch between the traditional and advanced techniques used for identifications. The current procedure, morphological and molecular identification, showed a high level of resemblance (97–100%). We obtained mitochondrial barcodes (543 and 519 bp) of *Cyt b* and 16S rRNA, respectively, for six fish species belonging to two families, two orders and five genera collected from six locations in the River Poonch (Figure 1). The amplified sequences did not contain any stop codons, insertions or deletion indicating that all of the segments represent functional mitochondrial sequences. All the fish species except one multiple specimen (minimum of three specimens per species) from different sampling sites were analyzed to document intraspecific divergence. Only one species, i.e., *G. kashmirensis,* were represented by single specimens (Table 1). As specimens were attested to species level based on phylogenetic trees, all morphological identifications improved with molecular identification since the specimens were attributed to species. However, Primers against *Cyt b* used in the present study yield an average nucleotide length of 543 bp, out of which 409 sites were constant, 50 showed singleton variables, 84 were parsimony informative sites and 134 were polymorphic. While in the case of 16S, rRNA 519 bp was obtained. Out of these, 399 sites were constant, 42 were singleton variable sites, 84 were parsimony informative sites, and 95 were polymorphic respectively. All these sequences were submitted to GenBank and got accession numbers presented in Table 1. Average nucleotide frequencies were A = 28.56%, T/U = 29.75%, C = 26.35% and G = 15.34% for *Cyt b*, while in case of 16S rRNA these were A = 32.39%, T/U = 21.22%, C = 24.51% and G = 21.88%, respectively.

As we expect, in a hierarchy rise in average genetic variant from within species (mean = 014%, standard error [SE] = .000, to within families (mean = .20%, SE = .001) was observed in the K2P model. As a whole, the genomic discrepancy among the same genus was roughly two times more than between the individuals of the same species. It was also noticed that the genetic distances between the lowest to highest taxonomic levels increased as the taxonomic levels increased as DNA sequence analysis of five fish species revealed that the intra-species genetic distance varied from .00 to .14. In contrast, the interspecies genetic distance ranged from .16 to .20. The maximum distance between *T. putitora* and *G. gotyla*, while minimum distances were observed between *T. putitora* and *T. latius*. However, in the case of 16S, the pairwise genetic distance for 16S rRNA sequencing depicted that the intraspecies genetic distance varied from .0 to .6. Similarly, the inter-species genetic distance was also noted from .08 to .16. The analysis also depicted that the maximum distance was observed for *G. kashmirensis* and *T. putitora,* while the minimum was for *S. plagiostomus* and *S. richardsonii* (Supplementary Tables S7, S8).

**FIGURE 6:**
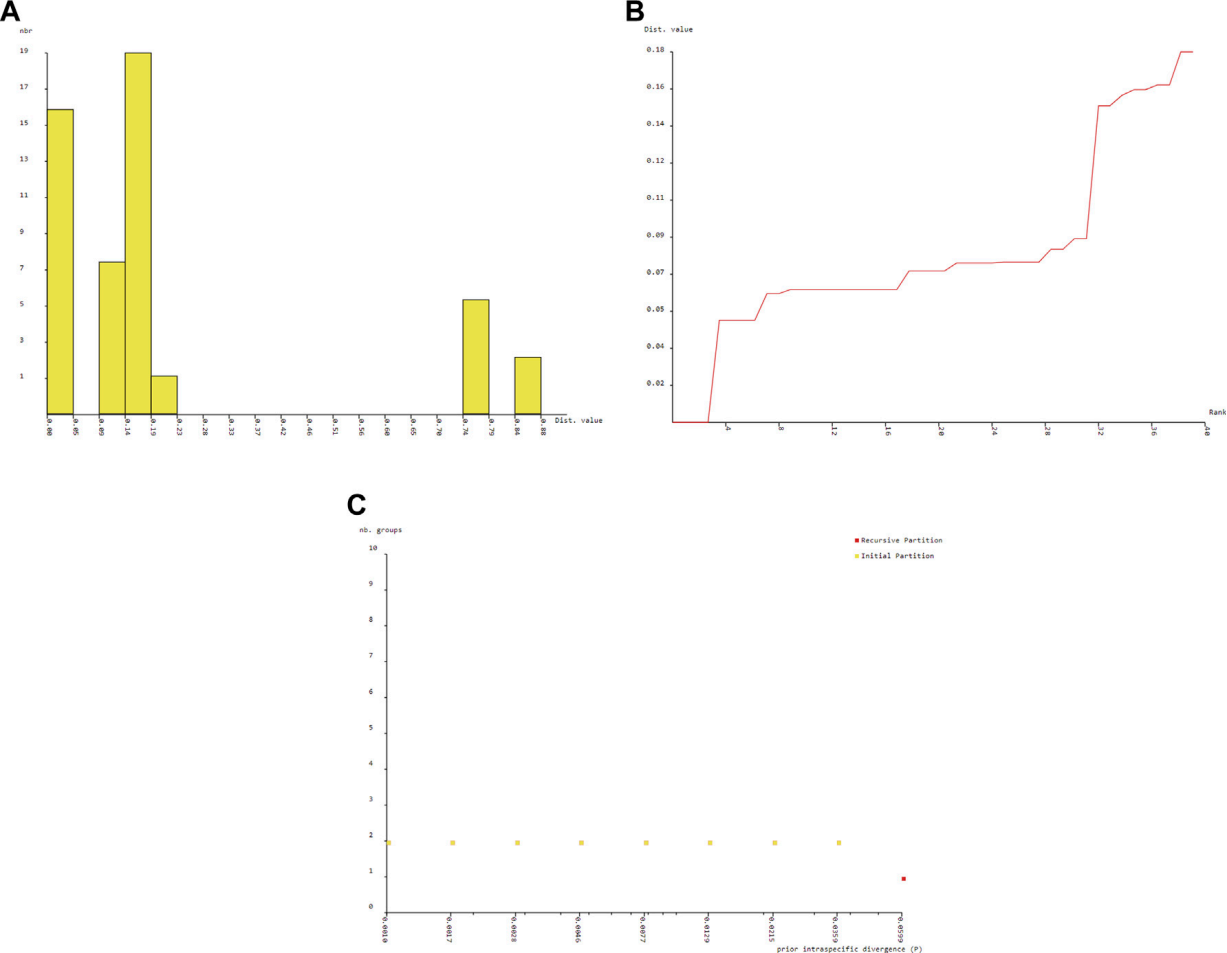
Barcode gap analysis of Pangasiid species generated by Automatic Barcode Discovery Gap Discovery (Puillandre et al., 2012). Distributions of K2P distances and between each pair of specimens **(A)** histogram of distance **(B)** ranked distance and **(C)** number of PSHs obtained for each prior intraspecific divergence.

The BIN analysis led to the recognition of 7 OTUs. All the BIN clusters were found to be taxonomically concordant with the other barcode that was BOLD assigned to the same species name. The count of OTUs produced by ABGD varied from 3 to 7 (Figure 6). The ABGD analysis conducted with K80 Kimura distance with Nb bins (for distance distribution) of 20 and gap widths (X) of .75 produced initial partitions with OUT count of 5 with Barcode gap distance = .069 for *Cyt b* (*p* = .021544–.012915), 3 in case of 16S rRNA (*p* = .021544–.012915) and 7 for combined CYT b +16S rRNA (*p* = .035938–.021544) respectively, whereas the use of P distance returned 5 (*p* = .021544–.012915), 3 (*p* = .021544–.012915) with Barcode gap distance = .493 and 7 (*p* = .035938–.021544) OTUs with a barcoding gap of .024. A comparison between the Bayesian inference and maximum-likelihood gene tree did not reveal the obvious difference in the positioning of OTUs. The two methods yielded congruent results with slight variation shown in Figure 6.

### Phylogenetic study

**FIGURE 7:**
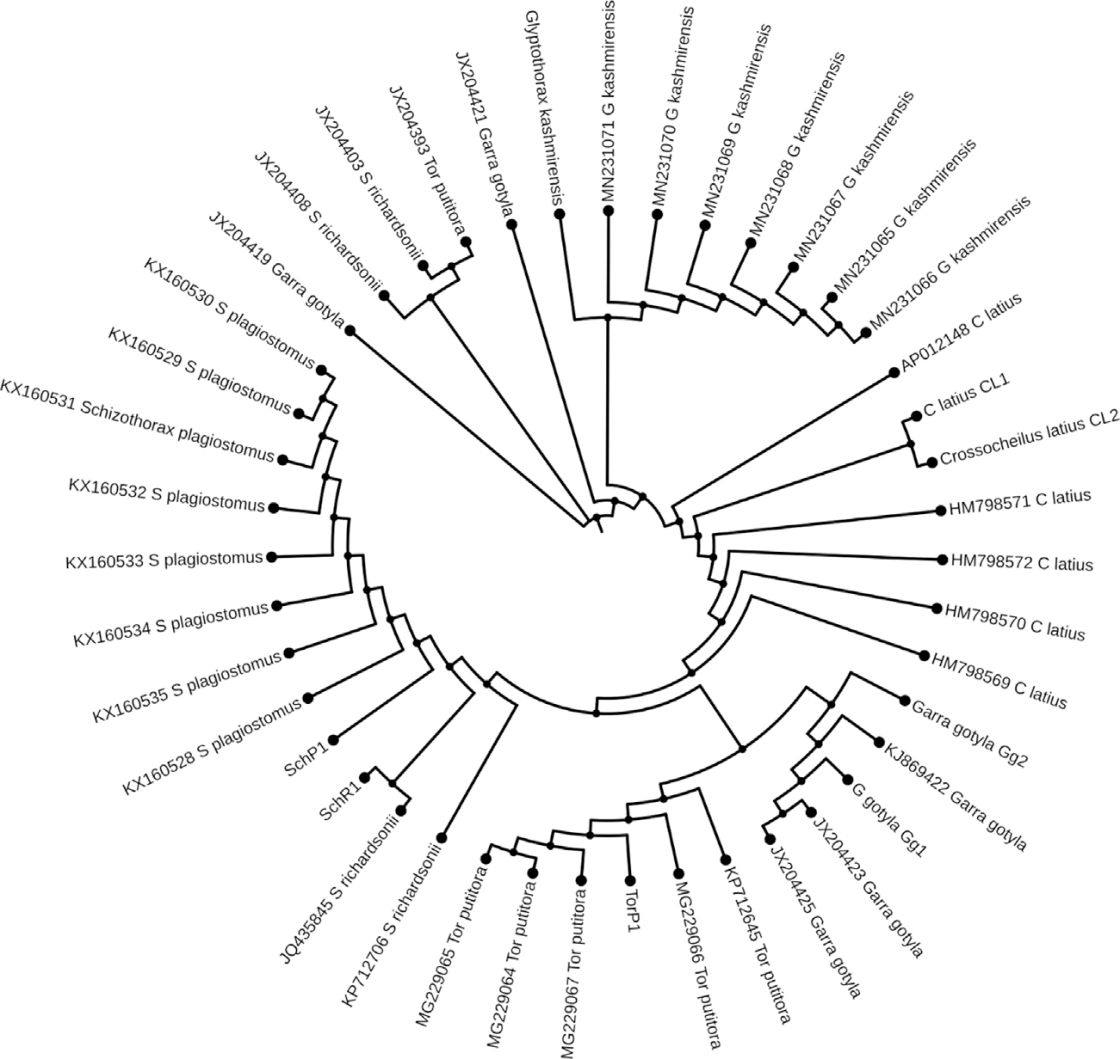
Maximum likelihood tree showing relationship of six fish species of River Poonch based on 16S rRNA data. The maximum likelihood tree was generated using GTR+G model.

**FIGURE 8:**
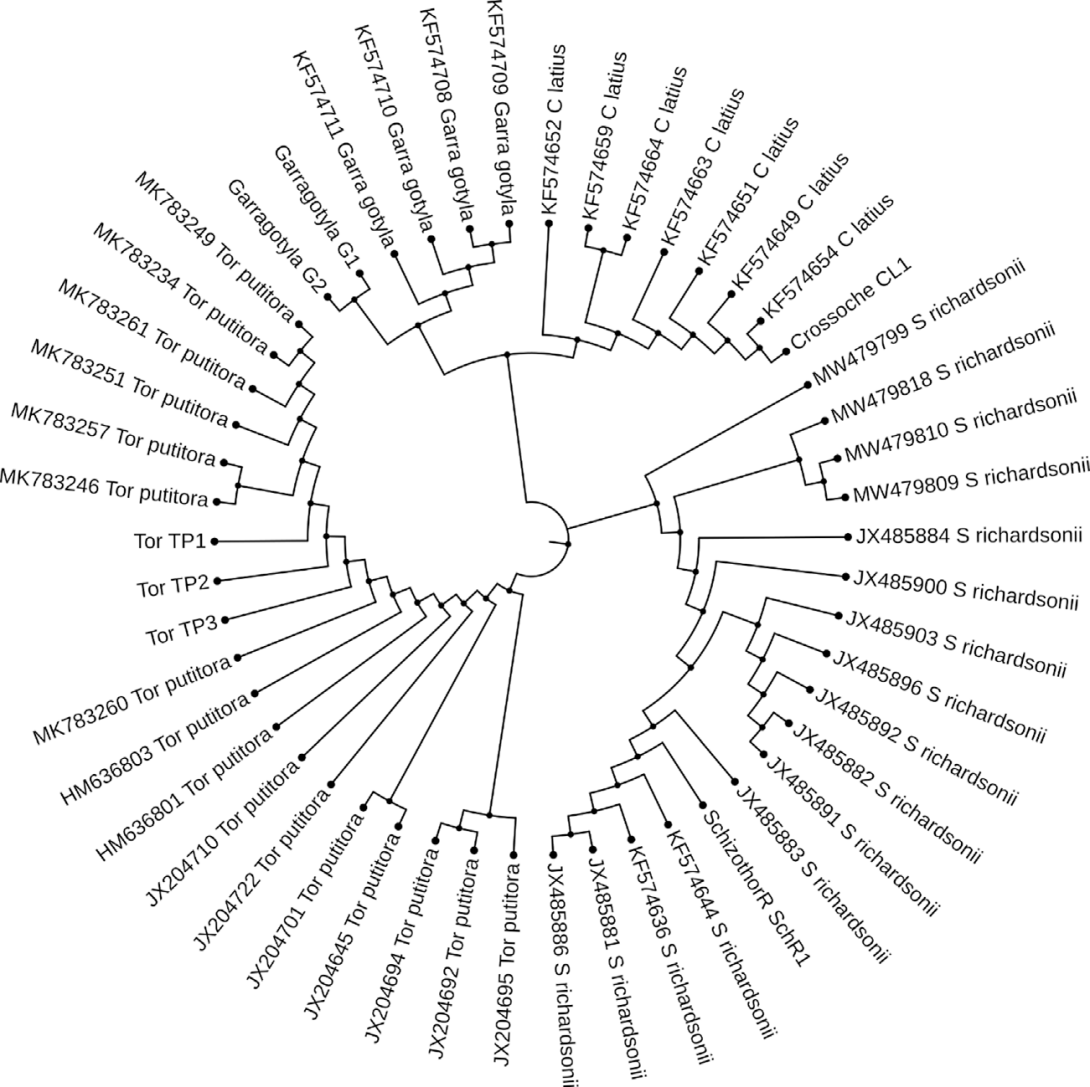
Maximum likelihood tree showing relationship of five fish species of River Poonch based on Cyt b data. The maximum likelihood tree was generated using GTR_G model.

**FIGURE 9:**
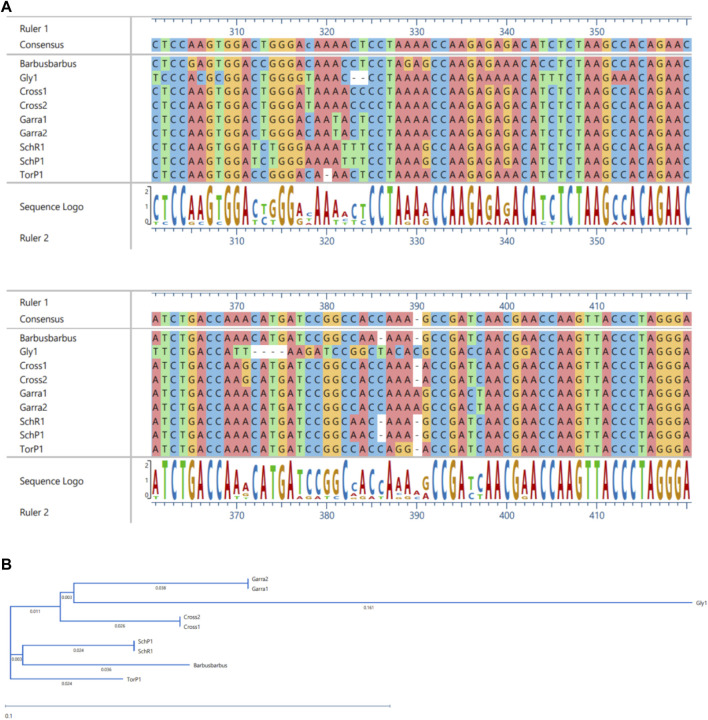
MSA **(A)** and Phylogenetic Tree **(B)** of 16S sequences. MSA was performed using MUSCLE Program and Phylogentic analysis were performed using PhyML algorithm (parameters—bootstrap value = 100, tree search = “Best from NNI and SPR,” Sarting tree = “BioNJ,” equllibrium and site rate variation = “Optimized”).

**FIGURE 10:**
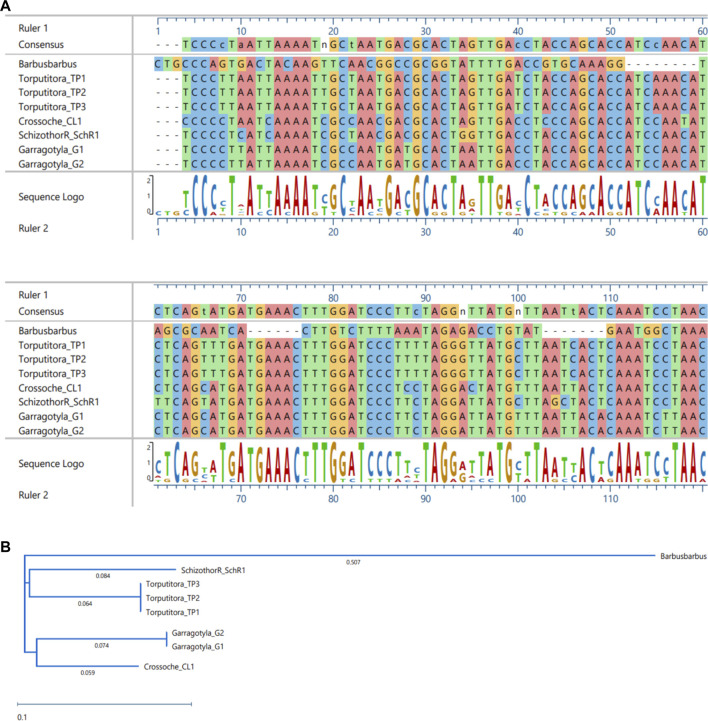
MSA **(A)** and Phylogenetic Tree **(B)** of CYT-B sequences. MSA was performed using MUSCLE Program and Phylogentic analysis were performed using PhyML algorithm (parameters—bootstrap value = 100, tree search = “Best from NNI and SPR”, Sarting tree = “BioNJ,” equllibrium and site rate variation = “Optimized”).

**FIGURE 11:**
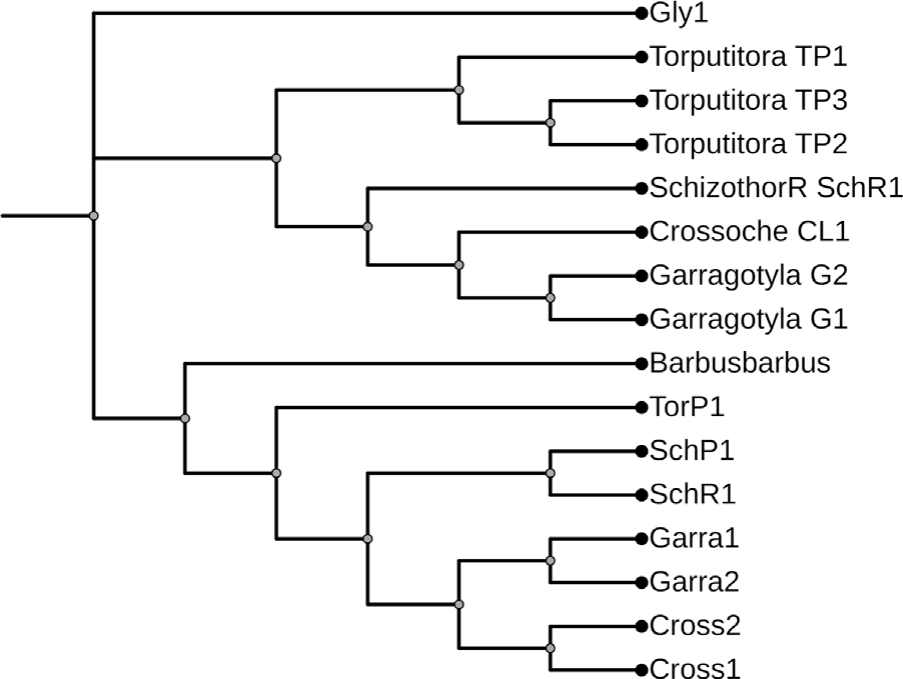
Maximum likelihood tree showing relationship of five fish species of River Poonch based on combine data. The maximum likelihood tree was generated using GTR+G model.

The ML and BI trees which were built based on the *Cyt b* and 16S rRNA dataset, placed the reference species grouped in close contact with the species identified by NCBI website, that proves the correctness of our morphological identification (Figures 7, 8). The ML and BI trees grouped sequences of the same taxonomically identified species indicated no overlapping clusters. The topology of ML and BI trees is almost identical. The topologies of *Cyt b* and 16S were slightly different; however, a better result was obtained through a combined approach and therefore, we describe it in detail (Figures 9–11). The result indicated that all six fish species were well differentiated according to their respective position. The tree topology for combined analysis (Figure 11) formed six monophyletic clad representing six species. According to their classification*, G. kashmirensis* form a separate clade, while other species form a clade and sub clade. The genus *Schizothorax* is placed within the same clade next to the *T. putitora*. While in the case of Tor, these are placed under the sister clade immediately next to the *Schizothorax* species.

On the other hand, *G. gotyla* and *T. latius* are placed under a sub-clade closer to the *Schizothorax* genus than *T. putitora* with BS = 81–100; PP = .76). Phylogenetic analyses were also performed separately for *Cyt b* and 16S using PhyML algorithm (parameters—bootstrap value = 100, tree search = “Best from NNI & SPR”, Starting tree = “BioNJ,” equilibrium and site rate variation = “Optimized”). The better result was obtained through *Cyt b,* in which five monophyletic clades were formed, while on the other hand, only three monophyletic clades were obtained for 16S. Moreover, similar results were obtained through incorporated data as all the species were grouped according to their classification, as shown in Figures 7, 8.

The haplotype networks of the closely related species demonstrated that the sharing of *Cyt b* and 16S rRNA haplotype was common for two genera (*Schizothorax* and *Tor*). The result of the current study noted four haplotypes with .644 haplotype diversity, while nucleotide diversity and overall mean distance were noted as .0904 and .24, respectively, for *Cyt b*. On the other hand, five haplotypes were observed with .899 haplotype diversity, while overall nucleotide diversity and mean found distance were recorded as .0720 and .08. The 16S rRNA sequencing helped us to estimate the average evolutionary divergence and overall sequence pair, which involved ten nucleotide sequences. The DNA sequence information of *Cyt b* gene’s able transversion/transition ratio basis (R) was observed as 3.219 and k1 = 4.17 for purine, while k2 = 7.78 for pyrimidines.

## Discussion

### Morphological characteristics

Poonch River is well known for its diverse fish fauna and serves as a lifeline for the large population. Over 25% population of the area is solely dependent on the fishery sector for their livelihood. The current study represents the first-ever effort to combine phenotypic and genotypic analysis of six economically important fish species inhabiting this Himalayan region (Poonch River). The multivariate analysis appears to be a practical approach for identifying and understanding evolutionary relationships among fish species. Therefore, it has gained importance in fishery research (Caillon et al., 2018; Behera et al., 2020). Cyprinid morphological variation, such as body profile, has rarely been studied (Bravi et al., 2013; Yan et al., 2013; Dwivedi et al., 2020). There are still some questions about the fish species inhabiting the Poonch River, and the criteria used to differentiate them have been limited to morphological and meristic characteristics (Day, 1878; Talwar and Jhingran, 1991; Jayaram, 2010). At first glance, after examining specimens morphologically, we were expecting nine fish species during the current study. But after integrative analysis, it was confirmed that only six fish species were there instead of nine. Because *G. gotyla* and *T. latius* were misidentified as *G. lamta* and *T. diplochelius,* respectively, as reported in the previous study by Dutta (2003). Whereas the genus Schizothorax could not be differentiated only on molecular analysis through an individual approach but combined with phenotypic study, one can easily distinguish these species (Lal et al., 2015; Dwivedi, 2020). On the other hand, after collection of Tor samples we were expecting two species, *viz.*, *T. putitora* and *Tor tor* as there was huge phenotypic ambiguity in the samples collected from the river, as by employing morphometric analysis it expressed some notable potential differences and on that basis one can easily consider them into two different species of Tor genus. Moreover, some distinguishing features between *T. putitora* and *T. tor* can be seen easily like head length, colour and size of barbells. But molecular results of the current study did not support the phenotypic results as after molecular analysis even not a single nucleotide difference can be sighted. So therefore, we can say that these morphological differences are not enough to surpass the molecular result. Hence through integrative approach we concluded that in Tor genus there is only one species i.e., *T. putitora* inhabiting River Poonch. However, such morphometric differences found in samples of Tor genus were to be only due to seasonal difference as the sample collected in different season (Pre-monsoon in which water is crystals clear (March) and during monsoon when the water is turbulent in the month of July as shown in Supplementary Figures S1A, B, respectively). The abovementioned results are in accordance with the findings of Naeem et al. (2011) and Langer et al. (2013), which suggested that *T. putitora* and *T. tor* are morphologically identical and are very difficult to differentiate without having proper taxonomic knowledge.

Moreover, in the current study, *G. lamta* and *T. diplocheilus* could not be differentiated by any approach; hence we conclude that these two species were misidentified from the region. However, Phenotypic analysis employing different approaches like PCA, CVA, RDA, correlation and dendrogram methods (Figures 2–4, 5A, B) among six fish species indicated an unambiguous correlation between the species based on their character. Additionally, three species of the genus Schizothorax and Tor (two and one species respectively) were a cluster in their relevant class, indicating that they share common phenotypic and body patterns and have similar body patterns. On the other hand, *G. kashmirensis* is placed distinctly from other fish species, which indicates that it differs in body profile and is characterized by a special set of features (Figure 3A). The present study’s results conform with the findings of other workers (Sarkar et al., 2012; Gupta et al., 2018; Dwivedi et al., 2020).

Based on the results, it was noted that out of six selected fish species, three species, *viz.*, *T. putitora*, *S. richardsonii* and *S. plagiostomus* have similar external morphological characteristics, making them difficult to distinguish. However, a lot of work has been carried out at the taxonomic level of these fish species, but still, there is uncertainty in the identification among these fish species (Pandey and Nautiyal, 1997; Kullander et al., 1999; Dwivedi et al., 2020; Pandey et al., 2020; Jaafar et al., 2021). The result indicated that all the parameters were associated with the total body length and head length. At the same time, variations observed in head length and fin rays became distinctive characteristic features for *T. putitora* (Figures 2, 5A, B). Our findings also revealed that *S. richardsonii* and *S. plagiostomus* are similar in appearance. Similar values were observed for all the parameters except maximum body depth, caudal fin length, and head length. Also, no differences in their habitat and spawning season were observed. The number of inferior pharyngeal teeth in both species was the same (4, 3, 2/2, 3, 4). A lot of work has been reported favoring the current results (Misra et al., 1980; Negi and Negi, 2010; Mir et al., 2013a; Wagle et al., 2015; Wani et al., 2018; Kamboj and Kamboj, 2019). Misra et al. (1980) revealed that both *S. richardsonii* and *S. plagiostomus* had the same number of teeth. Only slight differences in the shape and size of the ceratohyal and epihyal were noted, with the epihyal of *S. richardsonii* being smaller than *S. plagiostomus.* Other similar observations regarding the morphological variations were noted by Wagle et al. (2015), who reported that for *S. richardsonii*, all the parameters were undoubtedly interrelated with total length and head length; the current study also obtained similar results (Figures 5A,B). Thus, it is concluded that the anal fin length and lips resolve the phenotypic problem of differentiating between these two species. Therefore, the anal fin length of *S. plagiostomus* distinguishes it from *S. richardsonii*, as the anal fin of *S. plagiostomus* lying flat and long enough to touch the base of the caudal fin in contrast to the anal fin of *S. richardsonii* which is small, and never touches the base of the caudal fin.

### Molecular analysis (*Cyt b* + 16S rRNA)

Our study marks the first comprehensive molecular evaluation of the six fish species in the Poonch River. In the current study, DNA barcoding was effective in identifying species and provided a straightforward identification system when a perfect match existed between the morphology-based taxonomy and genetic divergence. Overall, this study demonstrated the ability of DNA barcoding to help calibrate the current taxonomic resolution and to shed new light on the fish diversity of River Poonch.

mtDNA is used for phylogenic surveys, genuine identification and differentiation of unidentified or closely linked species of aquatic organisms, including marine and freshwater fishes. A lot of work has been reported in the past on the taxonomy of these fish species from various parts of the world (Dimmick and Edds, 2002; He and Chen, 2006; Thai et al., 2007; Bajpai and Tewari, 2010; Kumar et al., 2011; Lakra et al., 2011; Yang et al., 2012; Ahmad et al., 2014; Chen et al., 2018; Krosch et al., 2019; Ma et al., 2020). However, it was challenging to compare our results with those who studied different species in a single attempt. No work has been reported on these fish species from this water body; however, little work has been reported from other water bodies in the region and other parts of the country (Ahmad et al., 2014; Jaafar et al., 2021), revealing the ambiguity among *Schizothorax* and *Tor* genus. But the current study is different from the previous ones (Ahmad et al., 2014) as they focused on the single genus of *Schizothorax*, but here we have adopted an integrative approach to five different genera, including *Schizothorax* and *Tor* as well, in a single attempt. Ghouri et al. (2020) reported the efficiency of *Cyt b* for the identification of edible fish species in Pakistan. Through the BLAST tool, they tracked similarities between species. In the current study, we used the ABGD, BINs, ML and BI approach and morphological study to authenticate fish species. However, compared to 16S rRNA, *Cyt b* is more successful in studying the intraspecies phylogenetic relationship. Besides molecular approaches, the morphological study made it more potent for validating the fish species of the area.

Genomics is valuable for preliminary species delimitations and for validating phenotypic-based species circumscription (Puillandre et al., 2012). At first glance, the analysis of *Cyt b* and 16S rRNA sequences with the genetic distance and topology created by the ML and BI tree discriminated all six fish species successfully as no overlapping clusters were formed. However, one species (*G. kashmirensis*) displayed deep divergence and two species of Schizothorax genera displayed the least divergence. A similar result was also found by using the ABGD method, in which the whole data set was delimited into six putative groups with a .24 barcoding gap. However, the individual phylogenetic trees showed overlapping in the case of the *Schizothorax*. *T. putitora, G. gotyla*, *T. latius*, and *G. kashmirensis* could be distinguished by all the methods like ABGD/ML analysis, even with an individual approach. However, the single-gene approach could not distinguish *S. richardsonii, S. plagiostomus*. The phylogenetic tree obtained by 16S rRNA showed a close relationship between the genus *Schizothorax* and formed only three monophyletic clades with three putative groups with three bins. On the other hand, in the case of *Cyt b,* there were five monophyletic clades formed with five putative groups in which five bins were present. However, in the case of *Cyt b*, better results were obtained as species of the Tor genus were partially differentiated. But in combined data that include both *Cyt b* and 16S in a single data set, all the species, including the genus of *Schizothorax*, could be distinguished. The current result was partially in accordance with the previous result of Ahmad et al. (2014), in which they emphasized more on combined analysis and got better results. However, in the case of our result, *G. gotyla* and *T. latius* shared a common sister clade and are also parallel with the result of Pandey et al. (2020), in which they found a similar type of result. Moreover, the current result indicated that the integrative approach could only be an effective tool to remove the ambiguity in these species.

During the current study, *G. gotyla*, *T. latius* and *G. kashmirensis* were clustered symmetrically in their respective genera. However, *G. gotyla* and *T. latius* were grouped and formed a separate sub-clade in all the cases, as both species belong to the sub-family Garrianae. Contrary to this, *G. kashmirensis* formed a separate clade both in individual (*Cyt b*) and (16S) as well as combined (*Cyt b* + 16S) phylogenetic analysis because of the difference in the family as it belongs to the family Sisordae. Conversely, all other species belong to the same family (Cyprinidae) and thus create a monophyletic clade according to their evolutionary history. But genus *Schizothorax* was not adequately differentiated with *Cyt b* as well as 16S individually. The current result was comparable with those of some earlier workers, as they have also noted the minimum genetic difference between these two species (Laskar et al., 2013; Khare et al., 2014; Yang et al., 2015; Laskar et al., 2018). In the present study, the phylogenetic results revealed a close relationship between *S. richardsonii* and *S. plagiostomus,* which supports the findings of other workers (Khan et al., 2016; Ma et al., 2020; Rehman et al., 2020). On the other hand, it has been noticed that there is a close association between the genus *Schizothorax* and *Tor* than *Garra* and *Tariqilabeo*. In general, similar species have been clustered together and different species have formed their cladogram (Lakra et al., 2011; Basheer et al., 2015; Bineesh et al., 2015; Davassy et al., 2015; Rathipriya et al., 2019). However, the considerable variability in their respective sites might be due to the *G. kashmirensis*, which belongs to Sisordae, whereas other species belong to the same family. Moreover, we found overall haplotype diversity higher in 16S rRNA compared to the *Cyt b* gene. In contrast, nucleotide diversity was noted more in the case of *Cyt b* than 16S rRNA. More haplotype diversity indicated that the diversity of this particular river is very high. Dekui et al. (2004) noted a similar result, who pointed out that the *Cyt b* gene revealed 659 constant sites, 481 variable sites, and 419 informative sites. The present results also showed similarity with the study of Lv et al. (2018), who obtained 42.7% of AT content during their analysis for fish species belonging to the family Sisordae.

For both genes, the analysis of the transition transversion ratio for all the species showed a higher transition ratio as compared to transversion, which is in agreement with the study of different authors (Page and Holmes, 1998; Ward et al., 2005; Li et al., 2013; Chakraborty et al., 2014; Bashir et al., 2016; Sharma et al., 2016). Our results also revealed that the average mean distance for *Cyt b* was noted in the range from .00 to .14 for intra-species, whereas .16 to .20 for interspecies with .01 standard error. The maximum distance was observed between *T. putitora* and *G. gotyla*, while the minimum was between *S. richardsonii* and *S. plagiostomus*. Comparable results were obtained from the 16S rRNA sequencing study, which revealed that within the species, variations varied from .00 to .6, but between species, variations ranged from .08 to .16. Moreover, the mean distance of individual species was noted as .00 to .041674 for *G. gotyla,* .00 to .026676 for *T. latius,* .00 to .018240 in the case of *S. plagiostomus* while for *S. richardsonii* it was varied from .00 to .027842, while, for *T. putitora* range from .00 to .022429 and for *G. kashmirensis* it was .075259. However, the extreme distance between *G. kashmirensis* and *T. putitora* was noted, while the least distance was observed for *S. plagiostomus* and *S. richardsonii*. The present study highlighted that intraspecies nucleotide diversity is far lower than inter-species genetic diversity, according to Ward et al. (2005) and Lakra et al. (2011). At the same time, Sherzada et al. (2020) also noted a gradual increase in genetic diversity with the rise in the genetic level. In the current study, the cases of weak genetic differentiation or haplotype sharing include four species from two genera. The accuracy of DNA barcoding to separate the species depends on the level of genetic diversity (Meyer and Paulay, 2005). Therefore, the incompetence to separate these species is credited to absence of sufficient genetic discrepancy between these species. In the genus *Schizothorax*, interspecific haplotype sharing is an omnipresent example in the same drainage (Dimmick and Edds, 2002; He and Chen, 2006). First, species inhabiting the same waterbody with a large allocation range can display structural variations like the shape of the mouth and lips in case of *Schizothorax*, head length, and body depth for *T. putitora* characteristics for identification (Wu and Wu, 1992; Chen et al., 2015).

## Conclusion

The integrative approach, i.e., morphometric and molecular phylogeny, is the most authentic and informative approach used to discriminate fishes (Rajpoot et al., 2016). In the current study, two mitochondrial genes were used to validate the fish species using a binary approach, such as phenotypic and genotypic data in order to resolve the ambiguity among six important fish species of the Poonch River. A combined analysis of mitochondrial loci mostly agrees with morpho taxonomical studies (Silas, 1960; Ahmad et al., 2014), indicating that the integrative method could effectively resolve uncertainty in the identification and understanding of morphological relationships even with a low sample size. Therefore, molecular phylogenies and phenotypic studies can be compared using standard methods to come up with a robust conclusion. The study showed that *Cyt b* and 16S rRNA were effective indicators of interspecies relationships and genetic variation. Although, we found that all other species, including *G. kashmirensis* (critically endangered), *T. putitora* (endangered), and *S. richardsonii* (vulnerable), are relatively large populations. However, they are still under intense pressure from human activities such as illegal fishing and killing species regardless of their conservation value.

## Data Availability

The datasets presented in this study can be found in online repositories. The names of the repository/repositories and accession number(s) can be found below: https://www.ncbi.nlm.nih.gov/genbank/, MW191577; https://www.ncbi.nlm.nih.gov/genbank/, MW191578; https://www.ncbi.nlm.nih.gov/genbank/, MW191579; https://www.ncbi.nlm.nih.gov/genbank/, MW191580; https://www.ncbi.nlm.nih.gov/genbank/, MW191581; https://www.ncbi.nlm.nih.gov/genbank/, MW191586; https://www.ncbi.nlm.nih.gov/genbank/, MW191585; https://www.ncbi.nlm.nih.gov/genbank/, MW148582; https://www.ncbi.nlm.nih.gov/genbank/, MW148583; https://www.ncbi.nlm.nih.gov/genbank/, MW148584; https://www.ncbi.nlm.nih.gov/genbank/, MW148588; https://www.ncbi.nlm.nih.gov/genbank/, MW148587; https://www.ncbi.nlm.nih.gov/genbank/, MW148589; https://www.ncbi.nlm.nih.gov/genbank/, MW148585; https://www.ncbi.nlm.nih.gov/genbank/, MW148586.

## Supplementary material

SUPPLEMENTARY FIGURE S1 Representative images of each species **(A)** *T. putitora* **(B)** *T. tor* **(C)** *G. gotyla* **(D)** *S. richardsonii* **(E)** *S. plagiostomus* **(F)** *T. latius* **(G)** *G. kashmirensis*.

SUPPLEMENTARY FIGURE S2 Location of 14 landmarks refers to 1. Anterior tip of snout at upper jaw 2 most posterior espect of neurocranium 3. Origin of dorsal; fin 4. Anterior attachment of dorsal membrane from caudal fin 5. Posterior end of vertebral column 6. Anterior attachment of ventral membrane from caudal fin 7. Origin of anal fin 8. Insertion of pelvic fin 9. Ventral edge of the operculum 10. Origen of pectoral fin 11. Posterior end of opperculum 12. Anterior end of eye 13. Posterior end of eye 14. Fork point of caudal fin.
