# Supplementary figures and images for: Integrative approach for validation of six important fish species inhabiting River Poonch of north-west Himalayan region (India)
Source: Front Genet. 2023 Jan 4;13:1047436. doi: 10.3389/fgene.2022.1047436 (PMC9886096; doi:10.3389/fgene.2022.1047436)

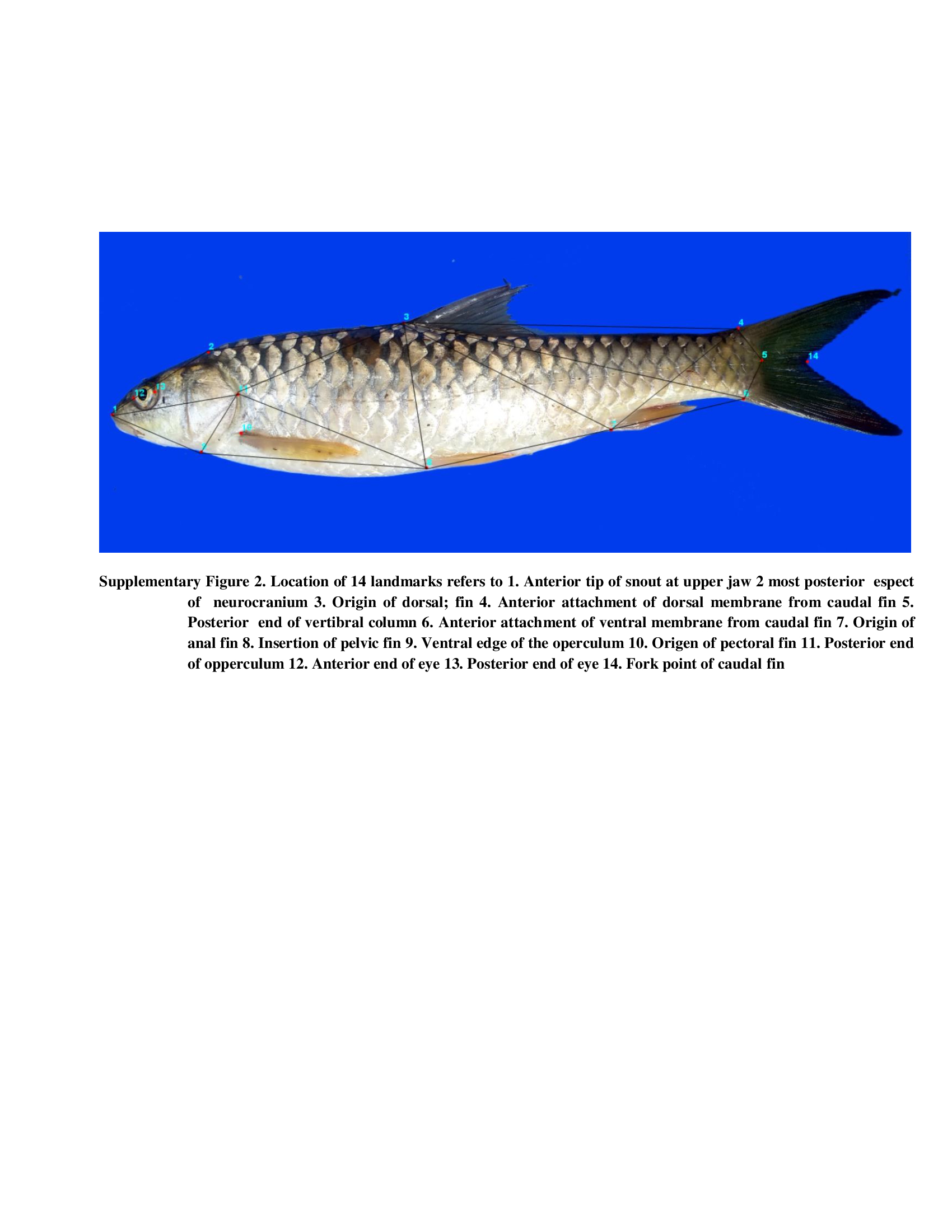

Supplement: Supplementary file 1 [file Image2.jpg]

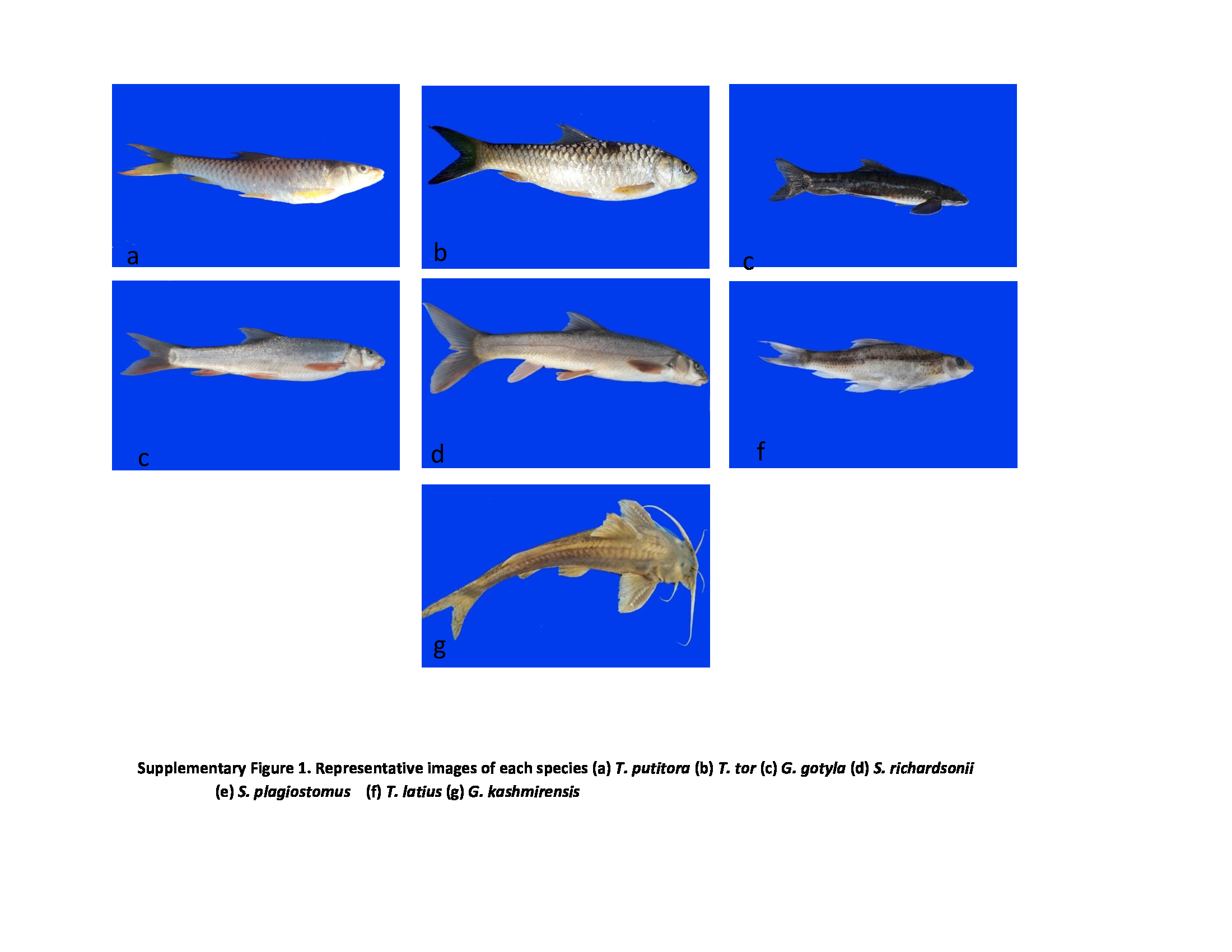

Supplement: Supplementary file 3 [file Image1.jpeg]
