# Supplementary material for: Integrative approach for validation of six important fish species inhabiting River Poonch of north-west Himalayan region (India)
Source: Front Genet. 2023 Jan 4;13:1047436. doi: 10.3389/fgene.2022.1047436 (PMC9886096; doi:10.3389/fgene.2022.1047436)
Supplement: Supplementary file 2 [file Table1.docx]

**Supplementary Table 1.** Correlation and regression analysis of morphometric characters of *T. putitora and T. tor* inhabiting the Poonch River.

|  | ***T. putitora*** | |
| --- | --- | --- |
| **Characters** | **Coefficient of correlation (r)** | **Regression equation**  **(Y = a + bX)** |
| Standard length | 0.98 | y=1.116 x-0.2508 |
| Fork length | 0.99 | y=1.102 x-0.195 |
| Pre pectoral length | 0.88 | y=0.883 x-0.458 |
| Pre pelvic length | 0.98 | y=1.241 x-.712 |
| Pre dorsal length | 0.96 | y=0.919 x-0.289 |
| Pre anal length | 0.99 | y=1.00 x-0.233 |
| Pectoral fin length | 0.45 | y=-0.1774 x+0.236 |
| Pectoral fin height | 0.78 | y=0.817 x-0.600 |
| Pelvic fin length | 0.52 | y=-0.461 x+0.61 |
| Pelvic fin height | 0.83 | y=0.861 x-0.692 |
| Dorsal fin length | 0.88 | y=0.779 x-0.652 |
| Dorsal fin height | 0.58 | y=0.348 x+0.101 |
| Anal fin length | 0.87 | y=0.806 x-0.964 |
| Anal fin height | 0.61 | y=0.428 x-0.147 |
| Caudal fin length | 0.98 | y=1.043 x-1.092 |
| Caudal fin height | 0.55 | y=0.415 x+0.056 |
| Maximum body depth | 0.56 | y=1.310 x-1.172 |
| Minimum body depth | 0.85 | y=1.151 x-1.264 |
| Snout length | 0.60 | y=0.769 x-0.292 |
| Eye diameter | 0.52 | y=0.572 x-0.434 |
| Pre orbital length | 0.77 | Y=1.070 X-0.512 |

**Supplementary Table 2.** Correlation and regression analysis of morphometric characters of *S*. *richardsonii* and *S. plagiostomus* inhabiting river Poonch

|  | ***S. richardsonii*** | | ***S. plagiostomus*** | |
| --- | --- | --- | --- | --- |
| **Characters** | **Coefficient of correlation (r)** | **Regression equation**  **(Y = a + bX)** | **Coefficient of correlation(r)** | **Regression equation**  **(Y = a + bX)** |
| Standard length | 0.98 | y=0.979 x-0.050 | 0.99 | y=1.00 x-0.088 |
| Fork length | 0.98 | y=1.039 x-0.093 | 0.99 | y=1.00 x-0.050 |
| Pre pectoral length | 0.84 | y=0.909 x-0.614 | 0.94 | y=0.946 x-0.650 |
| Pre pelvic length | 0.96 | y=0.952 x-0.307 | 0.52 | y=0.432 x+0.358 |
| Pre dorsal length | 0.88 | y=0.913 x-0.258 | 0.59 | y=0.596 x+0.164 |
| Pre anal length | 0.97 | y=0.905 x-0.797 | 0.93 | y=0.863 x-0.039 |
| Pectoral fin length | 0.59 | y=0.539 x-0.828 | 0.82 | y=1.139 x-1.620 |
| Pectoral fin height | 0.69 | y=0.752 x-0.543 | 0.85 | y=1.208 x-1.157 |
| Pelvic fin length | 0.68 | y=1.142 x-1.600 | 0.69 | y=1.034 x-1.420 |
| Pelvic fin height | 0.78 | y=1.141 x-1.098 | 0.89 | y=0.989 x-0.884 |
| Dorsal fin length | 0.80 | y=1.016 x-0.961 | 0.89 | y=1.099 x-1.112 |
| Dorsal fin height | 0.64 | y=0.576 x-0.241 | 0.72 | y=0.859 x-0.654 |
| Anal fin length | 0.69 | y=1.307 x-1.618 | 0.54 | y=1.091 x-1.318 |
| Anal fin height | 0.74 | y=0.996 x-0.859 | 0.74 | y=0.816 x-0.623 |
| Caudal fin length | 0.86 | y=1.145 x-1.174 | 0.57 | y=0.707 x-0.572 |
| Caudal fin height | 0.80 | y=0.835 x-0.493 | 0.89 | y=0.937 x-0.6139 |
| Maximum body depth | 0.58 | y=1.079 x-1.040 | 0.66 | y=1.175 x-0.935 |
| Minimum body depth | 0.60 | Y=0.671 X-0.620 | 0.73 | y=0.809 x-0.598 |
| Snout length | 0.59 | Y=0.557 X-0.115 | 0.49 | y=-0.084 x+0.166 |
| Eye diameter | 0.67 | Y=0.485 X-0.397 | 0.44 | y=-0.141 x-0.040 |
| Pre orbital length | 0.49 | Y=1.120 x-0.145 | 0.52 | Y=0.006 x+0.142 |

**Supplementary Table 3.** Descriptive statistics of different morphometric characters of *T. putitora*, *S. richardsonii,* and *S. plagiostomus* inhabiting the Poonch River.

|  | | ***S. richardsonii*** | | ***S. Plagiostomus*** | | | ***T. putitora*** | |
| --- | --- | --- | --- | --- | --- | --- | --- | --- |
| **S. No** | **In proportion to total fish length** | **Range** | **Mean±SD** | **Range** | | **Mean±SD** | **Range** | **Mean±SD** |
| 1. | Standard length | 10.32-31.76 | 21.1±3.2 | 14.2-28.9 | | 17.8±3.47 | 17.1-28.8 | 20.7±3.7 |
| 2. | Fork length | 13.37-34.54 | 23.3±3.8 | 15.5-32.9 | | 19.58±3.92 | 18.2-29.8 | 22.7±3.9 |
| 3. | Pre pectoral length | 3.93-7.23 | 4.6±0.8 | 3.1-6.5 | | 4.09±1.80 | 4.2-6.5 | 5.1±0.7 |
| 4. | Pre pelvic length | 15.87-7.43 | 10.8±1.6 | 6.1-14.7 | | 8.9±1.96 | 8.7-14.2 | 10.8±1.5 |
| 5. | Pre dorsal length | 14.76-6.89 | 10.6±1.7 | 7.1-12.7 | | 9.1±1.60 | 8.2-14.1 | 10.2±1.5 |
| 6. | Pre anal length | 20.56-11.94 | 15.6±2.1 | 10.5-19.7 | | 12.9±2.3 | 12.2-19.7 | 15.9±2.3 |
| 7. | Pectoral fin length | 0.5-1.3 | 1.2±0.1 | 0.9-1.5 | | 1.4±0.9 | 0.9-1.9 | 1.3±0.2 |
| 8. | Pectoral fin height | 2.6-4.5 | 3.3±0.7 | 1.7-4.5 | | 2.8±0.7 | 2.9-4.3 | 3.8±0.5 |
| 9. | Pelvic fin length | 0.6-1.7 | 1.3±0.3 | 0.8-2.5 | | 1.9±0.8 | 0.8-1.7 | 1.3±0.2 |
| 10. | Pelvic fin height | 2.5-4.7 | 3.2±0.7 | 2.9-5.7 | | 3.9±0.9 | 2.7-4.6 | 3.9±0.4 |
| 11. | Dorsal fin length | 1.9-4.2 | 2.9±0.5 | 1.7-4.9 | | 2.6±0.6 | 2.5-3.9 | 2.9±0.3 |
| 12. | Dorsal fin height | 2.8-5.2 | 3.8±0.5 | 1.4-4.9 | | 3.2±0.7 | 3.4-4.6 | 3.9±0.3 |
| 13. | Anal fin length | 1.1-2.1 | 1.7±0.4 | 1-2.9 | | 12.1±0.8 | 1.5-2.6 | 2.1±0.4 |
| 14. | Anal fin height | 2.8-5.4 | 3.5±0.7 | 2.6-5.7 | | 3.7±0.9 | 2.3-3.9 | 2.8±0.2 |
| 15. | Caudal fin length | 1.5-3.5 | 2.8±0.6 | 1.5-4.6 | | 2.9±0.9 | 2.3-3.9 | 2.7±0.4 |
| 16. | Caudal fin height | 3.7-7.2 | 4.7±0.8 | 3.3-6.1 | | 4.3±0.8 | 4.1-5.2 | 4.7±0.7 |
| 17. | Maximum body depth | 3.8-7.5 | 4.8±1.6 | 2.9-6.9 | | 4.3±1.8 | 2.9-6.9 | 5.2±1.7 |
| 18. | Minimum body depth | 1.9-3.5 | 2.8±0.7 | 1.6-3.9 | | 2.6±1.4 | 2.1-3.8 | 2.7±0.6 |
| 19. | Snout length | 1.2-2.7 | 1.7±0.7 | 1.5-2.9 | | 1.3±0.5 | 1.9-3.2 | 2.5±0.5 |
| 20. | Eye diameter | 0.5-1.2 | 0.9±0.09 | 0.7-1.1 | | 0.8±0.08 | 0.8-1.4 | 0.9±0.05 |
| 21. | Pre orbital length | 1.1-2.7 | 2.3±1.6 | 1.1-2.9 | | 2.6±0.8 | 1.9-2.9 | 2.1±0.3 |
| 22 | Head length | 3.3-5.9 | 3.9±0.6 | 2.9-5.7 | | 3.5±1.06 | 4.2-7.1 | 6.4±0.9 |
| Meristic Characters | | | | | | | | |
| 23 | Dorsal fin rays | 6-9 | | | 6-9 | | 8-11 | |
| 24 | Pect fin rays | 12-16 | | | 12-16 | | 10-14 | |
| 25 | Pelvic fin rays | 9-11 | | | 9-11 | | 8-9 | |
| 26 | Anal fin rays | 5-6 | | | 5-6 | | 5-6 | |
| 27 | Caudal fin rays | 16-18 | | | 16-18 | | 16-18 | |

**Supplementary Table 4.** Descriptive statistics of different morphometric characters of *T. latius* and *G. gotyla* inhabiting the Poonch River

|  | | ***T. latius*** | | ***G. gotyla*** | |
| --- | --- | --- | --- | --- | --- |
| **S. No** | In proportion to total fish length | Range | Mean ± SD | Range | Mean±SD |
| 1. | Standard length | 11.1-16.2 | 13.41±1.67 | 12.2-18.5 | 15.60±1.50 |
| 2. | Fork length | 12.2-18.1 | 14.68 ±1.92 | 13.2-20 | 17.05±1.51 |
| 3. | Pre pectoral length | 2.1-4.2 | 2.67± 0.53 | 2.5-4 | 3.10±0.37 |
| 4. | Pre pelvic length | 5.8-8.4 | 6.84±0.81 | 6.9-10 | 8.12±0.76 |
| 5. | Pre dorsal length | 5.3-8.1 | 6.02±0.87 | 6.7-8.5 | 7.44±0.59 |
| 6. | Pre anal length | 8.5-12.5 | 10.22±1.23 | 9.7-14.2 | 12.09±1.14 |
| 7. | Pectoral fin length | 0.9-2.9 | 1.97±0.63 | 1.1-1.6 | 1.29±0.13 |
| 8. | Pectoral fin height | 2.1-2.6 | 2.32±0.15 | 2.4-3.8 | 2.99±0.41 |
| 9. | Pelvic fin length | 0.5-0.9 | 0.76±0.13 | 0.8-1.9 | 1.09±0.28 |
| 10. | Pelvic fin height | 2.1-2.7 | 2.37±0.21 | 2.3-3.7 | 2.84±0.38 |
| 11. | Dorsal fin length | 1.8-2.6 | 2.17±0.25 | 2.4-3.3 | 2.73±0.24 |
| 12. | Dorsal fin height | 2.5-3.2 | 2.72±0.24 | 2.5-3.7 | 2.90±0.35 |
| 13. | Anal fin length | 0.9-1.5 | 1.45±0.21 | 1.1-2.1 | 1.47±0.27 |
| 14. | Anal fin height | 1.7-2.4 | 2.03±0.23 | 2.2-3.5 | 2.69±0.40 |
| 15. | Caudal fin length | 1.2-1.7 | 1.46±0.16 | 1.7-2.3 | 1.92±0.19 |
| 16. | Caudal fin height | 2.6-4.1 | 3.34±0.48 | 2.8-4 | 3.26±0.38 |
| 17. | Maximum body depth | 3.1-4.7 | 3.98±0.43 | 1.5-4.9 | 3.33±1.34 |
| 18. | Minimum body depth | 1.3-2.9 | 1.76±0.76 | 1.7-2.5 | 2.07±1.04 |
| 19. | Snout length | 1.2-1.9 | 1.75±0.23 | 1.2-2.5 | 1.96±0.43 |
| 20. | Eye diameter | 0.8-1.1 | 0.93±0.12 | 0.6-0.9 | 0.74±0.16 |
| 21. | Pre orbital length | 1.2-2.1 | 1.98±0.19 | 2-5 | 3.7±1.09 |
| 22 | Head length | 2.5-3.2 | 2.98±0.19 | 2.9-3.7 | 3.31±0.81 |
| **Meristic characters** | | | | | |
| 23 | Dorsal fin rays | 8-10 | 6-9 | | |
| 24 | Pect fin rays | 12-14 | 12-14 | | |
| 25 | Pelvic fin rays | 8-9 | 8-9 | | |
| 26 | Anal fin rays | 5-6 | 6-7 | | |
| 27 | Caudal fin rays | 16-20 | 16-18 | | |

**Supplementary Table 5.** Correlation and regression analysis of morphometric characters of *T.* *latius and G. gotyla* inhabiting the Poonch River

|  | ***T. latius*** | | ***G. gotyla*** | |
| --- | --- | --- | --- | --- |
| **Characters** | **Coefficient of correlation (r)** | **Regression equation**  **(Y = a + bX)** | **Coefficient of correlation (r)** | **Regression equation**  **(Y = a + bX)** |
| Standard Length | 0.95 | y=0.9002x+0.0309 | 0.80 | y=0.6771 x+0.2302 |
| Fork length | 0.97 | y=0.9489 x+0.0106 | 0.79 | y=0.7204 x-0.3274 |
| Pre pectoral length | 0.38 | y=07967 x-0.5478 | 0.17 | y=0.4186 x-0.0355 |
| Pre pelvic length | 0.62 | y=0.6915 x-0.0075 | 0.94 | y=0.7869 x-0.078 |
| Pre dorsal length | 0.40 | y=0.5977 x-0.0506 | 0.95 | y=0.891 x-0.189 |
| Pre anal length | 0.94 | y=0.8578 x-0.0352 | 0.77 | y=0.733 x+0.1623 |
| Pectoral fin length | 0.45 | y=-2.0767 x-2.5741 | 0.32 | y=0.5162 x-0.5365 |
| Pectoral fin height | 0.10 | y=-0.1586 x+0.5586 | 0.05 | y=0.2749 x+0.1274 |
| Pelvic fin length | 0.02 | y=0.0198 x-o.2048 | 0.03 | y=-0.369 x+0.4882 |
| Pelvic fin height | 0.65 | y=0.5421x-o.285 | 0.05 | y=0.2582 x+0.1271 |
| Dorsal fin length | 0.90 | y=0.8385 x-0.6845 | 0.52 | y=0.5554 x-0.2609 |
| Dorsal fin height | 0.68 | y=0.5352 x-0.216 | 0.06 | y=0.2436 x+0.1557 |
| Anal fin length | 0.53 | y=1.0718 x-1.2799 | 0.02 | y=-0.1226 x+0.3153 |
| Anal fin height | 0.09 | y=0.0719 x+0.2034 | 0.03 | y=-o.0832 x+0.5296 |
| Caudal fin length | 0.53 | y=0.6261 x-0.5978 | 0.46 | y=o.5284 x-0.3793 |
| Caudal fin height | 0.59 | y=0.8353 x-0.5115 | 0.63 | y=0.7969 x-0.4878 |
| Maximum body depth | 0.18 | y=0.3552 x+0.1132 | 0.61 | y=3.133 x-3.4438 |
| Minimum body depth | 0.81 | y=1.518 x-1.6112 | 0.38 | y=0.6137 x-0.4555 |
| Snout length | 0.99 | y = 0.4108x - 0.4202 | 0.1066 | y = -1.0843x + 0.8444 |
| Eye diameter | 0.25 | y = 1.7886x - 0.9252 | 0.0224 | y = 0.5758x - 0.5696 |
| Pre orbital length | 0.28 | y = 1.342x - 0.4033 | 0.1431 | y = -1.8982x + 1.4391 |

**Supplementary Table 6.** Correlation and regression analysis of morphometric characters of *G. kashmirensis* inhabiting the Poonch River

| ***G. kashmirensis*** | | | | |
| --- | --- | --- | --- | --- |
| **Characters** | **Range** | **Mean ± SD** | **Coefficient of correlation (r)** | **Regression equation (Y = a + bX)** |
| Standard length | 6.9-11.2 | 9.41±1.67 | 0.94 | y=0.9002x+0.0309 |
| Fork length | 4.8-9.8 | 07.68 ±1.92 | 0.95 | y=0.9489 x+0.0106 |
| Pre pectoral length | 2.1-4.2 | 2.67± 0.53 | 0.67 | y=07967 x-0.5478 |
| Pre pelvic length | 5.8-8.4 | 6.84±0.81 | 0.85 | y=0.6915 x-0.0075 |
| Pre dorsal length | 5.3-8.1 | 6.02±0.87 | 0.45 | y=0.5977 x-0.0506 |
| Pre anal length | 8.5-12.5 | 10.22±1.23 | 0.94 | y=0.8578 x-0.0352 |
| Pectoral fin length | 0.9-2.8 | 1.97±0.63 | 0.55 | y=-2.0767 x-2.5741 |
| Pectoral fin height | 2.1-2.6 | 2.32±0.15 | 0.34 | y=-0.1586 x+0.5586 |
| Pelvic fin length | 0.7-1.2 | 0.76±0.13 | 0.45 | y=0.0198 x-o.2048 |
| Pelvic fin height | 2.1-2.7 | 2.37±0.21 | 0.65 | y=0.5421x-o.285 |
| Dorsal fin length | 1.8-2.0 | 2.17±0.25 | 0.90 | y=0.8385 x-0.6845 |
| Dorsal fin height | 2.5-3.2 | 2.72±0.24 | 0.70 | y=0.5352 x-0.216 |
| Anal fin length | 0.9-1.5 | 1.45±0.21 | 0.58 | y=1.0718 x-1.2799 |
| Anal fin height | 1.7-2.4 | 2.03±0.23 | 0.34 | y=0.0719 x+0.2034 |
| Caudal fin length | 1.2-1.9 | 1.46±0.16 | 0.53 | y=0.6261 x-0.5978 |
| Caudal fin height | 2.6-4.1 | 3.34±0.48 | 0.59 | y=0.8353 x-0.5115 |
| Maximum body depth | 2.6-3.7 | 3.98±0.43 | 0.29 | y=0.3552 x+0.1132 |
| Minimum body depth | 1.3-2.3 | 1.76±0.76 | 0.81 | y=1.518 x-1.6112 |
| Snout length | 1.1-1.6 | 1.75±0.23 | 0.99 | y = 0.4108x - 0.4202 |
| Eye diameter | 0.4-0.6 | 0.38±0.12 | 0.25 | y = 1.7886x - 0.9252 |
| Pre orbital length | 1.2-2.1 | 1.98±0.19 | 0.45 | y = 1.342x - 0.4033 |
| Head length | 2.5-3.2 | 2.98±0.19 | 0.99 | y = 0.4108x - 0.4202 |
| **Meristic characters** | | | | |
| Dorsal fin rays | 8-10 | | | |
| Pectoral fin rays | 12-14 | | | |
| Pelvic fin rays | 8-9 | | | |
| Anal fin rays | 5-6 | | | |
| Caudal fin rays | 16-20 | | | |

**Supplementary Table 7.** Genetic divergence analysis of 16S rRNA gene of seven fish species of river Poonch.

|  | **B. barbus** | **Gly1** | **C1** | **C2** | **G1** | **G2** | **SR1** | **SP1** | **TP1** |
| --- | --- | --- | --- | --- | --- | --- | --- | --- | --- |
| ***B. barbus*** |  |  |  |  |  |  |  |  |  |
| **Gly1** | 0.161 |  |  |  |  |  |  |  |  |
| **C1** | 0.084 | 0.166 |  |  |  |  |  |  |  |
| **C2** | 0.084 | 0.166 | 0.000 |  |  |  |  |  |  |
| **G1** | 0.089 | 0.185 | 0.064 | 0.064 |  |  |  |  |  |
| **G2** | 0.089 | 0.185 | 0.064 | 0.064 | 0.000 |  |  |  |  |
| **SR1** | 0.064 | 0.166 | 0.077 | 0.077 | 0.078 | 0.078 |  |  |  |
| **SP1** | 0.064 | 0.166 | 0.077 | 0.077 | 0.078 | 0.078 | 0.000 |  |  |
| **TP1** | 0.062 | 0.158 | 0.063 | 0.063 | 0.072 | 0.072 | 0.048 | 0.048 |  |

Gly1= *G. kashmirinsis*, C1+C2= *C.latius*, G1+G2= *G. gotyla*, SR1= *S. richardsonii*, SP1= *S. plagiostomus* and TP1= *T. Putitora*

**Supplementary Table 8.** Genetic divergence analysis of Cyt b gene of five fish species of river Poonch.

|  | ***B. barbus*** | **TP1** | **TP2** | **TP3** | **CL1** | **SR1** | **G1** | **G2** |
| --- | --- | --- | --- | --- | --- | --- | --- | --- |
| ***B. barbus*** |  |  |  |  |  |  |  |  |
| **TP1** | 0.191 |  |  |  |  |  |  |  |
| **TP2** | 0.191 | 0.000 |  |  |  |  |  |  |
| **TP3** | 0.191 | 0.001 | 0.001 |  |  |  |  |  |
| **CL1** | 0.188 | 0.141 | 0.141 | 0.141 |  |  |  |  |
| **SR1** | 0.180 | 0.179 | 0.179 | 0.179 | 0.183 |  |  |  |
| **G1** | 0.198 | 0.159 | 0.159 | 0.159 | 0.140 | 0.200 |  |  |
| **G2** | 0.198 | 0.159 | 0.159 | 0.159 | 0.140 | 0.200 | 0.000 |  |

**Supplementary Table 9.** classical taxonomy of seven fish species habiting river Poonch

| **Species** | **Family** | **Classical taxonomy** |
| --- | --- | --- |
| ***G. gotyla*** | Cyprinidae | The chin has a suctorial disc; the snout has a well-developed median proboscis and a transverse lobe at the tips; the snout has prominent tubercles; the snout has two pairs of barbels (maxillary and rostral); the head, dorsally and flanked, is dark brownish; the ventral part of the body is light grey; the lateral line is complete |
| ***T. putitora*** | Cyprinidae | lateral line scale with 27-30; dark greenish dorsally; yellowish gold flank; silvery/ white ventrally; body elongated and laterally compressed; head length considerably more than body depth at dorsal fin origin. |
| ***S. richardsonii*** | Cyprinidae | Long, subcylindrical, and somewhat compressed body; Body dark grey with black speckles on top and silvery underneath. Fins have a light golden; The snout is pointed and blunt; lower jaw is large, posteriorly positioned, and highly cornified, with a keratinized cutting edge and a continuous lower labial fold. Two pairs of barbels, maxillary and rostal; 8-rayed dorsal fin; 15–16 rays on the pectoral fin; 8 rays on the pelvic fin; 5 rays on the anal fin; Caudal fin forked; Minute scale |
| ***S. plagiostomus*** | Cyprinidae | Silvery colour with a few small black dots; body is elongate and subcylindrical. The snout is sharp and sleek, and the head is small. The mouth is subterminal, arched, and has thick, flashing lips. The size of the eye diameter is less important than the number of barbells. Dorsal fin with 11 rays; Pectoral fin with 17 rays; pelvic fin with 8 rays; anal fin with 8 rays, caudal 11-12 rays. |
| ***T. latius*** | Cyprinidae | The body is elongated grey with irregular patches of black markings, and the eyes are located below the head's centre. A frenulum links the top lip to the lower jaw, making the mouth inferior. There are two sets of barbells, with the maxillary being smaller than the eye diameter. The entire body is covered with medium scales. The dorsal fin has 11 rays, the pectoral fin has 13-14 rays, the pelvic fin has 9 rays, the anal fin has 7 rays, and the caudal fin has 18-20 rays**.** |
| ***G. kashmirensis*** | Sisordae | Stretched body with a somewhat golden ventral side and a grey dorsal side. The lips are pipillated and the mouth is inferior with four pairs of barbels. The species' distinguishing characteristic is a longitudinal striated thoracic adhesive organ with a central spheroid pit.; Dorsal fin with 5-6 rays; Pectoral fin with 8-9 rays; anal fin with 8 rays, caudal 16-18 rays; |
